# The *parABS_m_* system is involved in megaplasmid partitioning and genome integrity maintenance in *Thermus thermophilus*

**DOI:** 10.1093/g3journal/jkad038

**Published:** 2023-02-14

**Authors:** Haijuan Li, Lingling Xu, Xiaoxiao Li

**Affiliations:** College of Biological and Environmental Engineering, Xi’an University, No. 168 South Taibai Road, 710065 Xi’an, China; College of Biological and Environmental Engineering, Xi’an University, No. 168 South Taibai Road, 710065 Xi’an, China; College of Biological and Environmental Engineering, Xi’an University, No. 168 South Taibai Road, 710065 Xi’an, China

**Keywords:** polyploid bacteria, *Thermus thermophilus*, megaplasmid, *parABS*, toxin–antitoxin system

## Abstract

The characteristics of the *parABS* system in polyploid bacteria are barely understood. We initially analyzed the physiological functions and mechanisms of the megaplasmid *parABS_m_* system in the thermophilic polyploid bacterium *Thermus thermophilus*. Deletion of *parAB_m_* was possible only when a plasmid-born copy of *parAB_m_* was provided, indicating that these genes are conditionally essential. The cell morphology of the *parAB_m_* deletion mutant (Δ*parAB_m_*) was changed to some extent, and in certain extra-large or twisted cells, the nucleoids were dispersed and damaged. Compared with that of the wild type, the frequency of anucleate cells was significantly increased. Genome content analyses showed that Δ*parAB_m_* had lost ∼160 kb of megaplasmid and ∼23 kb of chromosomal sequences, respectively. Genome fluorescent tagging and PFGE experiments demonstrated that the truncated megaplasmid was frequently interlinked and could not be segregated correctly; thus, certain daughter cells eventually lost the entire megaplasmid and became twisted or enlarged with damaged nucleoids. Further, we found that when the megaplasmid was lost in these cells, the toxins encoded by the megaplasmid toxin–antitoxin (TA) systems (VapBC64_65 and VapBC142_143) would exert detrimental effects, such as to fragment DNA. Thus, *parABS_m_* might ensure the existence of these TA systems, thereby preventing genomic degradation. Together, our results suggested that in *T. thermophilus*, the megaplasmid-encoded *parABS* system plays an essential role in the megaplasmid partitioning process; also it is an important determination factor for the genome integrity maintenance.

## Introduction

Plasmids are ubiquitous in prokaryotic bacteria and play essential roles in cell metabolism, pathogenesis, and species evolution. Naturally occurring plasmids come in different sizes, namely, plasmids (small, usually 10^3^–10^5^ bp, including multicopy nonconjugative plasmids and low-copy conjugative plasmids), megaplasmids (the thresholds for minimum megaplasmid size are difficult to be defined, but they are normally >5% genome size), and “chromids” (different from megaplasmid and frequently encode essential genes) (Hall *et al*. 2022). How plasmids can be stably maintained with a constant copy number in host cells is of great interest for microbiologists. It is suggested that plasmid positioning and segregation mode depend on their copy numbers (Salje 2010; Million-Weaver and Camps 2014; Baxter and Funnell 2014). Plasmids with high copy number tend to segregate randomly in a passive way (Nordström and Gerdes 2003; Million-Weaver and Camps 2014; Reyes-Lamothe *et al*. 2014). By contrast, low-copy-number plasmids (normally 1–2 copies of plasmid per cell, e.g. F and P1 plasmids in *Escherichia coli*) have evolved active partitioning systems to ensure their faithful inheritance to daughter host cells (Million-Weaver and Camps 2014; Baxter and Funnell 2014; Bouet and Funnell 2019). These plasmid partitioning systems are usually consisted of three elements: one or several copies of *cis*-acting site (centromere-like sequence area), one centromere-binding protein (adaptor protein), and one protein-encoding ATPase or GTPase (motor protein) (Million-Weaver and Camps 2014; Bouet and Funnell 2019). The adaptor and motor protein-encoding genes are arranged in one operon and auto-regulated; the centromeric sequence is often positioned near the operon and is composed of tandem repeat (direct or inverted) sequences (Million-Weaver and Camps 2014; Hu *et al*. 2017; Bouet and Funnell 2019). For low-copy-number plasmids, disruption of either one or three of these partitioning factors would cause segregation defects, thus yielding plasmid loss at high rates (Ebersbach and Gerdes 2001; Li *et al*. 2004).

Based on the enzyme activity type of the motor protein, the plasmid partitioning systems have been classified into three types (Bouet and Funnell 2019). The most prevalent one is the type I systems which feature a Walker-type ATPase, for example, the well-studied *parABS* system of the *E. coli* P1 plasmid (Gerdes *et al*. 2000; Bouet and Funnell 2019). In *parABS*, *parS* is the centromere site, ParA is the ATPase, and ParB is the *parS*-binding protein. When the *parABS* system starts to partition plasmids, ParB binds *parS* and extends to neighboring DNA forming a large nucleoprotein complex, while ParA proteins are polymerized to filaments. The ParA filaments can contact the ParB–*parS* complex, once upon ATP hydrolysis, they will rapidly disassemble, thereby pulling plasmids to the quarter-cell position prior to cell division (Gerdes *et al*. 2000; Baxter and Funnell 2014; Hu *et al*. 2017; Bouet and Funnell 2019). Type II and type III plasmid partitioning systems employ actin-like ATPase and tubulin-like GTPase as the motor proteins, respectively, and also work via a dynamic motor protein polymerization mechanism (Møller-Jensen *et al*. 2002; Larsen *et al*. 2007; Bouet and Funnell 2019). Besides being present in the low-copy-number plasmids, homologs of the type I system (i.e. the *parABS* system) were later found in two-thirds of bacterial chromosomes that have been sequenced (Gerdes *et al*. 2000; Jalal and Le 2020). However, in contrast to the plasmid counterpart, the chromosomally encoded *parABS* system has pleiotropic functions (Murray and Errington 2008). For example, they were shown to be involved in chromosome replication and segregation in *Bacillus subtilis* (Lee and Grossman 2006); initiation of cytokinesis and regulation of DNA replication initiation in *Caulobacter crescentus* (Mohl *et al*. 2001; Murray and Errington 2008); cell growth, chromosome segregation, and cell motility in *Pseudomonas aeruginosa* (Bartosik *et al*. 2004; Lasocki *et al*. 2007); and cell morphology maintenance in *Pseudomonas putida* (Lewis *et al*. 2002). Further, the participation of the chromosomal *parABS* orthologs in the chromosome segregation process is not as significant as that of their plasmid equivalents (Badrinarayanan *et al*. 2015). Although deletion of the chromosomal *parAB* genes in some bacterial species (e.g. *B. subtilis* and *P. aeruginosa*) could elevate number of anucleate cells, most cells still exhibited normal chromosome segregation (Lee and Grossman 2006; Lasocki *et al*. 2007). In some cases, mutation of these genes would not cause cell growth defect or increasement of anucleate cells (e.g. in *Vibrio cholerae* and *Thermus thermophilus*) (Fogel and Waldor 2006; Li *et al*. 2015). Therefore, it is currently explained that the chromosomal *parABS* systems mainly function in the segregation of origin-proximal regions but not of bulk chromosomes (Badrinarayanan *et al*. 2015).

Besides low-copy-number plasmids and bacterial chromosomes, certain bacterial megaplasmids also encode the *parABS* orthologs (e.g. *Deinococcus radiodurans* and *T. thermophilus*); however, their physiological functions and mechanisms were nearly unknown (Li *et al*. 2015; Maurya *et al*. 2019). In recent decade, a series of bacteria were determined to be polyploid, which means one single cell contains multiple copies (>3) of chromosome (Soppa 2014). How these multiple genome copies can be partitioned to the daughter cells (i.e., via a stringent or a random pattern) remains unknown. Moreover, whether the *parABS* orthologs in these genomes play roles in their vertical transmission also remains to be mysterious. *T. thermophilus* is able to grow from 50°C to 80°C, and due to its various merits, it has been established as a model organism for studying thermophilic bacteria. The genome of *T. thermophilus* HB27 (NCBI: GCA_000008125.1) is constituted by a chromosome (1.89 Mb) and a megaplasmid (0.27 Mb), which are both shown to be present with 4–5 copies per cell under slow growth condition (Ohtani *et al*. 2010; Li 2019a). Both the chromosome and megaplasmid encode the *parABS* orthologs (termed as *parABS_c_* and *parABS_m_*, respectively). The megaplasmid also encodes two sets of toxin–antitoxin (TA) loci (*vapBC* belonging to type II TA), which normally function to prevent the survival of plasmid-free cells by a post-segregational killing mechanism or play roles in response to cellular stresses (Kamruzzaman *et al*. 2021). The TA systems are composed of two genes encoding toxin and antitoxin, respectively; the antitoxins act as antidotes for the toxins. When both genes were disrupted, the concentrations of antitoxins in the cells would be rapidly decreased due to their instability property (Kamruzzaman *et al*. 2021). The stable and unopposed toxins would then exert their toxic effects leading to plasmid loss or cell death (Bardaji *et al*. 2019; Kamruzzaman *et al*. 2021). The toxins can kill the cells by targeting a variety of important cellular processes, including cytoskeleton formation and cell-wall synthesis, membrane integrity, DNA replication, transcription, and translation (Fraikin *et al*. 2020). VapBC comprises the largest TA family, and its toxin VapC normally functions as a nuclease which may have different target specificities in different organisms (Arcus *et al*. 2011; Fan *et al*. 2017).

Our previous studies showed that the chromosomal-encoded *parABS_c_* system was not required for the chromosome segregation (Li *et al*. 2015). The megaplasmid *parAB_m_* genes were undeletable; through partially knocking down the genes, we found that the *parAB_m_* knockdown mutant demonstrated growth and nucleoid defects and deletion of a portion of the megaplasmid sequences (Li *et al*. 2015). Therefore, we hypothesized that if the *parAB_m_* genes were completely deleted, there might be more detrimental effects for the cells, such as deletion of larger portion of the genome (including the megaplasmid and chromosome) or loss of the entire megaplasmid, nucleoid fragmentation, cell morphology variation, cell death, and severe megaplasmid segregation defect. Also, it is interesting to reveal the mechanism about how the *parABS_m_* system functions to maintain the megaplasmid. On the basis of our previous research, in this study, we detailedly addressed the physiological role and working mechanism of the megaplasmid-encoded *parABS_m_* system. We found that complete deletion of *parAB_m_* genes could cause severe cell growth defect and yield deletion of megaplasmid and chromosome sequences. A fraction of Δ*parAB_m_* mutant cells became enlarged or twisted and with aberrant and fragmented nucleoids, in which the truncated megaplasmid was frequently mislocalized. TUNEL (TdT-mediated dUTP nick-end labeling) assay showed that this kind of cells was mostly dead cells. We further proved that deletion of *parAB_m_* could eventually lead to loss of the entire megaplasmid in these aberrant cells, thereby triggering TA system-mediated nucleoid degradation and cell death. Together, it was concluded that in *T. thermophilus*, the *parABS_m_* system is not only essential for the megaplasmid partitioning but also critical for the genomic sequence and structure integrity maintenance.

## Materials and methods

### Bacterial strains and growth conditions

*E. coli* DH5a was the host strain for constructing all the plasmids and was grown in LB medium under 37°C. The wild-type (WT) *T. thermophilus* strain (HB27, DSM7039) and its derivative strains were grown in TB medium at 70°C or 60°C. TB medium was composed of 4 g/l yeast extract, 8 g/l trypticase peptone (BD Biosciences), and 3 g/l NaCl, and the pH value was adjusted to 7.5. The growth media were supplemented with kanamycin (50 μg/ml and 20 μg/ml for *E. coli* and *T. thermophilus*, respectively), bleomycin (Sigma-Aldrich, 15 μg/ml for *E. coli* and 3 μg/ml for *T. thermophilus*), or nalidixic acid (Nal) (100 μg/ml) when necessary.

### Plasmid constructions

All plasmids and strains used in this study are summarized in Table 1, and the oligonucleotides used for PCR amplification are listed in Supplementary Table 1. The allele exchange vectors pUC-Δ*parAB_m_::blm*, pUC::Δ*parB_c_::kat-parB_c__sgfp*, pUC::Δ*fdh::kat-parB_m__sgfp*, pUC-Δ*vapBC64_65::kat*, and pUC-Δ*vapBC142_143::blm* were derived from the fundamental vector pUC18. For pUC-Δ*parAB_m_::blm*, pUC-Δ*vapBC64_65::kat*, and pUC-Δ*vapBC142_143::blm*, the thermostable antibiotic genes *kat* and *blm* were, respectively, PCR amplified from pMK18 and pMB18 vectors (*E. coli*/*T. thermophilus* shuttle vectors) used in former studies (de Grado *et al*. 1999; Brouns *et al*. 2005; Li *et al*. 2015). Approximately 1 kb upstream and downstream flanking regions of the *parAB_m_*, *vapBC64_65*, or *vapBC142_143* locus were PCR amplified from *T. thermophilus* HB27 (GCA_000008125.1), respectively. The primers used in these reactions would confer the PCR fragments 20-bp sequences overlapping pUC18 and *kat*/*blm*; thereby, the recombinant vectors could be constructed by fragment in-fusion (i.e. Gibson assembly) method (Gibson *et al*. 2009) (reagents were from New England Biolabs). For constructing pUC::Δ*parB_c_::kat-parB_c__sgfp* and pUC::Δ*fdh::kat-parB_m__sgfp*, pMK-*parB_c__sgfp* and pMK-*parB_m__sgfp* were constructed first (the schematic designs of the constructs are shown in Supplementary Fig. 1), for which *parB_c_* and *parB_m_* were, respectively, PCR amplified and translationally fused with the *sgfp* gene of the pMK-*sgfp* vector used in a previous study (Li *et al*. 2015). The *kat-parB_c__sgfp* and *kat-parB_m__sgfp* fragments were then amplified from pMK-*parB_c__sgfp* and pMK-*parB_m__sgfp* and cloned into the middle of the two flanking homology regions (around 1 kb) of *parB_c_* and *fdh* (TTP0138, encoding formate dehydrogenase) under the pUC18 background, resulting pUC::Δ*parB_c_::kat-parB_c__sgfp* and pUC::Δ*fdh::kat-parB_m__sgfp*, respectively. In these two vectors, *parB_c__sgfp* and *parB_m__sgfp* were both transcribed under the *slp* promoter (i.e. promoter of the *kat* gene). The complementation vector pMK-*parAB_m_* was derived from pMK18. In detail, *parAB_m_* was PCR amplified and transcriptionally fused to the *kat* cassette of pMK18 using the same Gibson assembly method (Gibson *et al*. 2009).

**Table 1.** Strains and plasmids used in this study.

Name	Description	Reference
**Plasmids**		
pUC-Δ*parAB_m_::blm*	Allele exchange vector for generating Δ*parAB_m_*, *ori* pUC, Blm^R^	This study
pUC::Δ*parB_c_::kat-parBc_sgfp*	Allele exchange vector for generating Δ*parB_c_::kat-parB_c__sgfp*, *ori* pUC, Kam^R^	This study
pUC::Δ*fdh::kat-parB_m__sgfp*	Allele exchange vector for generating Δ*fdh::kat-parB_m__sgfp*, *ori* pUC, Kam^R^	This study
pUC-Δ*vapBC64_65::kat*	Allele exchange vector for generating Δ*vapBC64_65::kat*, *ori* pUC, Kam^R^	This study
pUC-Δ*vapBC142_143::blm*	Allele exchange vector for generating Δ*vapBC142_143::blm*, *ori* pUC, Blm^R^	This study
pMK18/pMB18	*E. coli*/*T. thermophilus* shuttle vectors, *T. thermophilus* (*repA*), *E. coli* (*oriE*), Kam^R^ (pMK18), or Blm^R^ (pMB18)	(de Grado *et al*. 1999; Brouns *et al*. 2005)
pMK-*sgfp*	Derived from pMK18, expressing sGFP under the *slp* promoter	(Li *et al*. 2015)
pMK-*parAB_m_*	Derived from pMK18, allowing overexpression of ParAB_m_ in *T. thermophilus*	This study
**Strains**		
HB27	*T. thermophilus* wild type	DSM7039
Δ*bgl*	*T. thermophilus* HB27 derivative with *bgl* gene clean deleted	(Angelov *et al*. 2013)
Δ*parAB_m_*	*T. thermophilus* HB27 derivative with *parAB_m_* deleted	This study
Δ*parB_c_::kat-parB_c__sgfp*	*T. thermophilus* HB27 derivative with *parB_c_* replaced by *kat-parB_c__sgfp*, expressing ParB_c_-sGFP	This study
Δ*fdh::kat-parB_m__sgfp*	*T. thermophilus* HB27 derivative with *fdh* replaced by *kat-parB_m__sgfp*, expressing ParB_m_-sGFP	This study
Δ*parAB_m_*Δ*parB_c_::kat-parB_c__sgfp*	*T. thermophilus* Δ*parAB_m_* derivative with *parBc* replaced by *kat-parB_c__sgfp*, expressing ParB_c_-sGFP	This study
Δ*parAB_m_*Δ*fdh::kat-parB_m__sgfp*	*T. thermophilus* Δ*parAB_m_* derivative with *fdh* replaced by *kat-parB_m__sgfp*, expressing ParB_m_-sGFP	This study
Δ*parAB_m_*/ParAB_m_	Δ*parAB_m_* derivative overexpressing ParAB_m_	This study
Δ*vapBC64_65::kat*	*T. thermophilus* HB27 derivative with *vapBC64_65* replaced by *kat*	This study
Δ*vapBC64_65*/*142_143*	*T. thermophilus* HB27 derivative with *vapBC64_65* replaced by *kat* and *vapBC142_143* replaced by *blm*	This study

### Mutant generations

For generation of *T. thermophilus* Δ*parAB_m_* mutant, the common method for deletion of bacterial essential genes was utilized (a schematic description of the mutant generation procedure is shown in Supplementary Fig. 2) (Kruse *et al*. 2005; Yamaichi *et al*. 2007). Initially, the *parAB_m_* gene deletion vector (pUC-Δ*parAB_m_::blm*, linearized by *Hind*III before using) and the exogenous plasmid encoding ParAB_m_ (pMK-*parAB_m_*) were co-transformed to the WT *T. thermophilus* cells (natural competent). After addition of DNA, the cells were exposed to appropriate conditions (70°C, 180 rpm/min) for 3 h to grow, also to absorb and recombine DNA. During this period, at first, the megaplasmid *parAB_m_* (including *parS_m_* on *parB_m_*) was still present in the cells, the megaplasmid/plasmid-expressed ParB_m_ would bind the *parS_m_* site, and ParA_m_ would also provide force for pushing/or pulling the ParB_m_–*parS_m_* complex. Since the copy number of pMK-*parAB_m_* could reach 4–10 copies/cell, in addition, it contained a strong promoter (*slp*) in front of the *parAB_m_* genes (de Grado *et al*. 1999), the ParAB_m_ proteins from which could be overexpressed. Constant overexpression of ParAB_m_ from the exogenous plasmid would automatically render the megaplasmid-encoded ParAB_m_ proteins unessential. Consequently, the antibiotic gene *blm* from the gene deletion vector pUC-Δ*parAB_m_::blm* would easily replace the megaplasmid *parAB_m_* genes. The transformation reaction was then spread on TB plate supplemented with kanamycin for the selection of cells containing pMK-*parAB_m_*. The colonies of the *parAB_m_* deletion mutant were distinguishable from other transformants which only contained pMK-*parAB_m_* and with the megaplasmid *parAB_m_* present, as they were apparently small and white (upon loss of the megaplasmid *parAB_m_*, a large portion of the genome sequences including the *blm* gene was sequentially lost, resulting this aberrant phenotypes, see results). Finally, to remove the pMK-*parAB_m_* vector from the *parAB_m_* deletion mutant cells, three white and small transformants were randomly picked and, respectively, inoculated in liquid TB medium without any selection and grown (70°C, 180 rpm/min) for 72 h, and then the cultures were probably diluted and plated on TB plate without any selection. Approximately 100 colonies of each transformant from these TB plates were picked and streaked on three types of plates, respectively, i.e. TB + kanamycin, TB + bleomycin, and TB. And streaks grown only on TB plate but not on the other two types of plates were defined as Δ*parAB_m_* mutants that have lost the pMK-*parAB_m_* vector. Three Δ*parAB_m_* streaks were then randomly selected for genotype confirmation by PCR (using two sets of primers binding the control gene and *parA_m_* or *parB_m_* genes, respectively) and/or genome sequencing. To confirm that it was the exogenously expressed ParAB_m_ proteins but not the shuttle vector (i.e. pMK18) itself that would recover the complete *parAB_m_* deletion mutant, we also co-transformed pUC-Δ*parAB_m_::blm* and pMK18 to the WT *T. thermophilus* cells followed by mutant selection using the similar method (a schematic illustration is shown in Supplementary Fig. 3).

For generation of Δ*vapBC64_65::kat*, the pUC-Δ*vapBC64_65::kat* vector was linearized by *Hind*III and transformed to the WT *T. thermophilus* cells. The transformants were selected on TB plate supplemented with kanamycin, and the genotype was confirmed by PCR using primers flanking the deletion region. For generation of the double-gene deletion mutant Δ*vapBC64_65*/*142_143*, the pUC-Δ*vapBC142_143::blm vector* (linearized by *Hind*III) was transformed to the Δ*vapBC64_65::kat* mutant cells, followed by selecting on TB + kanamycin + bleomycin plate. The mutant genotype was also confirmed by PCR. The generation procedures of Δ*parB_c_::kat-parB_c__sgfp*, Δ*fdh::kat-parB_m__sgfp*, Δ*parAB_m_*Δ*parB_c_::kat-parB_c__sgfp*, and Δ*parAB_m_*Δ*fdh::kat-parB_m__sgfp* are shown in Supplementary Fig. 1.

### Analyses of DNA content

PCR for determining the megaplasmid and chromosome sequence loss was performed based on standard protocol, except that two pairs of primers were used in one reaction, one pair of which was amplifying the target locus, and the other was amplifying the control gene region [i.e. TTC0825 (734 bp) or TTC1220 (890 bp) for detecting megaplasmid deletion and TTC0439 (857 bp) for detecting chromosome deletion]. β-Glucosidase (Bgl) activity assay was performed according to the method described in Li *et al*. (2015), for which three independently grown cell cultures were used. The quantitative PCR (qPCR) experiment for assessing the relative megaplasmid copy number was essentially performed as described previously (Breuert *et al*. 2006). Two megaplasmid loci (TTP0108 and TTP0161) were selected as the qPCR amplification targets, and one chromosomal locus near the *oriC* (TTC1609) was chosen as the internal reference amplification region (the primers used are listed in Supplementary Table 1). The amplicons were between 100 and 200 bp; three biological and three technical repeats were carried out for each strain. The relative quantification 2^−ΔΔCt^ method was used to calculate the relative (to WT) megaplasmid copy number of the Δ*parAB_m_* or Δ*parAB_m_*/ParAB_m_ strain. For genome sequencing, genomic DNA of the Δ*parAB_m_* or Δ*vapBC64_65*/*142_143* mutant strain was respectively isolated by Monarch Genomic DNA Purification Kit (New England Biolabs) based on the protocol provided by the manufacturer, and the purified gDNA (suitable for Next Generation Sequencing) was sequenced by BGI Technology (Shenzhen, China). The following genome sequence alignment method for determining genome deletion in the mutants was essentially based on previous studies (Bardaji *et al*. 2019; Ramijan *et al*. 2020). Specifically, the CLC Genomics Workbench software (8.5.1) was then used to analyze the Illumina reads. Raw Illumina reads of the Δ*parAB_m_* or Δ*vapBC64_65*/*142_143* mutant were imported and mapped to the reference genome of *T. thermophilus* HB27 (NCBI reference sequence: GCA_000008125.1) via the “Map reads to reference” function in the NGS core tools. Pulsed-field gel electrophoresis (PFGE) was essentially performed as described by Herschleb *et al*. (2007). The WT and Δ*parAB_m_* strains were grown to exponentially growing phase, and the cell pellets were collected by centrifugation at 4,000 rpm/min for 20 min, respectively. The cell-holding agarose plugs were prepared using SeaKem Gold agarose (Lonza). The genomic DNA in the plugs was directly exposed for electrophoresis without digestion. One percent of SeaKem Gold agarose prepared in 0.5 × TBE was used for gel casting. The electrophoresis was carried out in a PFGE CHEF-DR III variable angle system (Bio-Rad), and the samples were run in the following conditions for 24 h: 120 degree included angle, 6 V/cm, 8–50 s switch time ramp, and 14°C.

### Analyses of nucleoid morphology and segregation

For analyzing cell and nucleoid morphology, exponentially growing cells were collected, and the cell pellets were resuspended in 1 × PBS. DAPI (4′,6-diamidino-2-phenylindole-dihydrochloride) and Neuro-DiO were then used to stain the nucleoid and cell membrane, with final concentrations of 1 and 10 μg/ml, respectively. After incubating at RT for 30 min, the residual dyes were washed by 1 × PBS for three times, and the cells were resuspended in the same buffer. An appropriate amount (10 μl) of the cells was then mounted on glass slides followed by microscopic analysis. For visualizing the chromosome and megaplasmid segregations of the WT and Δ*parAB_m_* cells, the ParB-*parS* fluorescent tagging system was used (Hu *et al*. 2007; Badrinarayanan *et al*. 2015). Specifically, the allele exchange vector pUC::Δ*parB_c_::kat-parB_c__sgfp* or pUC::Δ*fdh::kat-parB_m__sgfp* was individually transformed into WT or Δ*parAB_m_*, respectively. The transformants were selected by kanamycin, and the correct recombinants were confirmed by PCR (with primers flanking the homology regions). In the correct recombinants (i.e. Δ*parB_c_::kat-parB_c__sgfp*, Δ*fdh::kat-parB_m__sgfp*, Δ*parAB_m_*Δ*parB_c_::kat-parB_c__sgfp*, or Δ*parAB_m_*Δ*fdh::kat-parB_m__sgfp*), the chromosomal *parB_c_* or the megaplasmid *fdh* gene was, respectively, replaced by *kat-parB_c__sgfp* or *kat-parB_m__sgfp* fusion genes (Supplementary Fig. 1). ParB_c_-sGFP and ParB_m_-sGFP were able to bind their cognate *parS_c_* and *parS_m_* sites, which were, respectively, residing in the chromosomal replication origin-proximal region and the megaplasmid *parB_m_* gene itself. Thus, the subcellular localization and segregation pattern of the ParB_c_-sGFP and ParB_m_-sGFP fluorescent signals reflected those of the chromosome and megaplasmid, respectively. The four strains expressing ParB_c_-sGFP or ParB_m_-sGFP were then grown to exponential phase, and the cells were collected for fluorescence microscopy. All the fluorescence microscopic experiments were performed with a Nikon Ni-U microscope. The cell length, width, the sizes of the DAPI-stained nucleoids, and the ParB_c_-sGFP/ParB_m_-sGFP foci numbers were all analyzed by using ImageJ software (NIH, USA). The nucleoid amount and nucleoid morphology of the cells were reflected by the dimension and pattern of the DAPI-stained area, respectively (Schneider *et al*. 2007). The significance of differences of these parameters between two strains was tested by Student's *t*-test (*P* < 0.01 indicates significant difference, *P* > 0.05 indicates no difference).

### TUNEL assay

Although TUNEL was initially used to analyze DNA fragmentation in eukaryotic cells, it was later also shown to be effective for detecting nucleoid damage and cell death in prokaryotic cells (Rohwer and Azam 2000). TUNEL is based on the principle that when DNAs are damaged, their free 3′-OH ends will be exposed, which can be enzymatically labeled with dUTP-fluorescein isothiocyanate by using terminal deoxynucleotidyl transferase (TdT). The nucleoids in the cells will thus be fluorescently labeled which can be easily detected using fluorescence microscopy or flow cytometry (Rohwer and Azam 2000). In our study, to assess DNA degradation and cell death of the Δ*parAB_m_*, Δ*vapBC64_65*/*142_143*, and Nal-treated cells, TUNEL assay was performed using the In Situ Cell Death Detection Kit, Fluorescein (Roche Applied Science), according to the protocol provided by the manufacturer. The fluorescein-dUTP labeled broken DNA of these cells was observed by fluorescence microscopy.

## Results

### Generation of *parAB_m_* gene deletion mutant

**Fig. 1.**
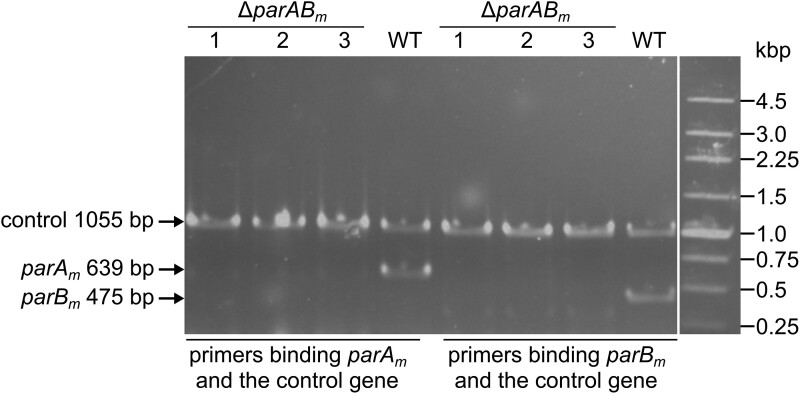
Generation of the *T. thermophilus* Δ*parAB_m_* mutant. The mutant was confirmed by PCR using two sets of primers binding the control gene and *parA_m_* or *parB_m_* genes, respectively. The control gene used in these PCR reactions was TTC0213 residing in the chromosome. The primer pairs used for amplifying *parA_m_*, *parB_m_*, and TTC0213 were dparA_m_-F and dparA_m_-R, dparB_m_-F and dparB_m_-R, and TTC0213-F and TTC0213-R, respectively (listed in Supplementary Table 1). 1, 2, and 3 represent three individual mutants. The 250 bp DNA ladder (Takara, Japan) was used for the agarose gel electrophoresis.

The chromosomally encoded *parABS* locus normally resides in the very vicinity of the replication origin region of the chromosome. Likewise, the *T. thermophilus* megaplasmid *parABS_m_* is also positioned near the megaplasmid replication origin region. *parABS_m_* is composed of *parA_m_* (TTP0084) and *parB_m_* (TTP0083) genes transcribed in one operon and a *parS_m_* sequence which is residing in the *parB_m_* gene itself (5′-AAGGACGCGTCCTT-3′) (Li *et al*. 2015). *T. thermophilus* is polyploid; when a gene is essential, the deletion mutant will remain heterozygous which contains both the WT and the mutant allele at the same gene locus (Ohtani *et al*. 2010; Li *et al*. 2015; Li and Gao 2021). Using a standard gene exchange method (replaced by a thermostable bleomycin resistance gene cassette *blm*), we found that the *parAB_m_* complete deletion mutant was impossible to be obtained (i.e. remained heterozygous) (Li *et al*. 2015), unless an exogenous plasmid (pMK18-*parAB_m_*) encoding the WT copy of ParAB_m_ was co-transformed with the gene deletion vector (pUC-Δ*parAB_m_::blm*) into the *T. thermophilus* cells (see *Materials and methods*). This result implied that both of the *parA_m_* and *parB_m_* genes are conditionally essential. Without antibiotic (kanamycin) selection, pMK18-*parAB_m_* could be easily lost from the host cells (the loss of the plasmid was confirmed by growth test of the mutant in TB medium supplemented with kanamycin), thereby resulting pure Δ*parAB_m_* mutant (see *Materials and methods*). PCR and genome sequencing results confirmed that the *parAB_m_* operon has been completely deleted in the Δ*parAB_m_* mutant (Fig. 1; Fig. 3c and d). It is worthy to note that, in the above experiment, it was the exogenously expressed ParAB_m_ proteins but not the shuttle vector (i.e. pMK18) itself that worked to recover the complete *parAB_m_* deletion mutant. As when we co-transformed pUC-Δ*parAB_m_::blm* and pMK18 to the WT *T. thermophilus* cells followed by the similar mutant selection procedure (Supplementary Fig. 3), no white transformant colonies were observed, which indicated that no large megaplasmid deletion had occurred (comparing with the phenotypes of the complete *parAB_m_* deletion mutant; see the following results). Further, PCR analysis confirmed that all the resulting *parAB_m_* deletion mutants were heterozygous (containing both WT-*parAB_m_* and Δ*parAB_m_::blm* alleles) but not complete deletion (Supplementary Fig. 4).

### Growth rate, cell, and nucleoid morphology of the *parAB_m_* mutant

**Fig. 2.**
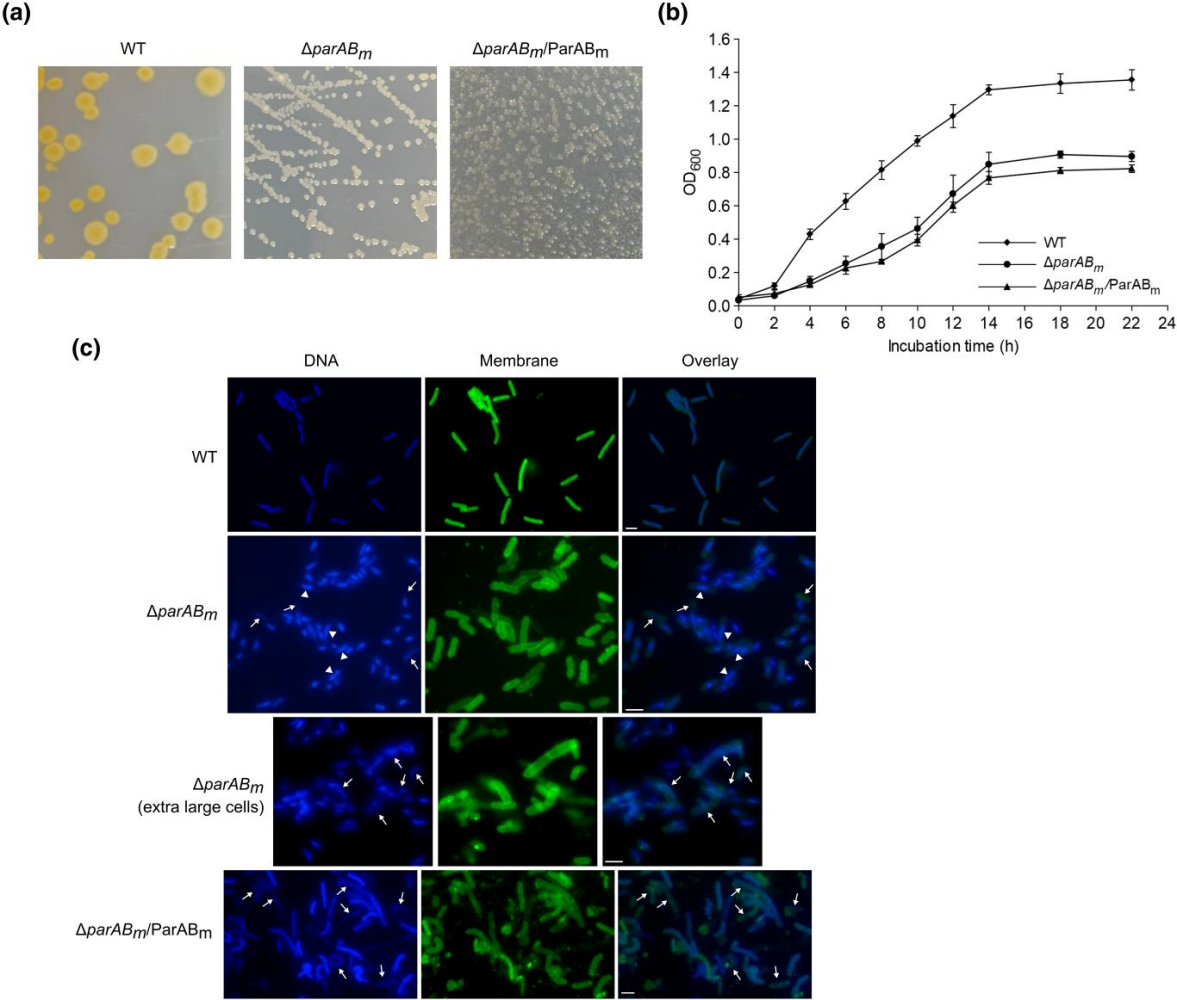
Growth rate, cell, and nucleoid morphology of the Δ*parAB_m_* mutant. a) Colony morphology of the wild-type strain (WT), the Δ*parAB_m_* mutant, and the Δ*parAB_m_*/ParAB_m_ strain. b) Cell growth curves of the three strains, mean, and standard deviations (SD) of the OD_600_ values from three independent measurements are shown. c) Cell and nucleoid morphology of the three strains. The membrane and nucleoid were stained by Neuro-DiO and DAPI, respectively (see *Materials and methods*). Dispersed or damaged nucleoids are pointed with arrows; unsegregated nucleoids are pointed with triangles; bars, 2 μm.

The Δ*parAB_m_* mutant exhibited small and white colonies which were strikingly different from the WT colonies (i.e. bigger and showing orange–yellow color) (Fig. 2a). The mutant cells also demonstrated severe growth defect when incubated in liquid medium (Fig. 2b), indicating that lack of *parAB_m_* affected the WT cell growth. Fluorescent microscopic analyses showed that the Δ*parAB_m_* cells were significantly (*P* < 0.01, 100 cells were counted for each strain) shorter and wider than the WT cells growing at a same growth phase (Fig. 2c; Table 2). Some of the mutant cells even became twisted or extraordinarily enlarged (on average 1.38 ± 0.29 times wider than the WT cells, 30 enlarged cells were counted for Δ*parAB_m_*, also 30 cells were counted for WT) (Fig. 2c), indicating that deletion of *parAB_m_* has directly or indirectly rendered cell morphology change. According to the dimension and pattern of the DAPI-stained areas (Schneider *et al*. 2007), the nucleoid amount and nucleoid morphology of the cells were analyzed. We found that although with swelled cell shape, the nucleoids in the mutant cells were smaller compared with those in the WT cells (100 cells were measured for each strain, *P* < 0.01). Further, most nucleoids in the mutant cells remained unsegregated in which the replicated nucleoids (represented by two DAPI-stained regions) remained together (Fig. 2c, examples are pointed by triangles), and the frequency of anucleate cells was increased to 8.5% (only 0.4% was detected in the WT cells, Table 2). Moreover, in the extra-large or irregularly shaped cells, the nucleoids were dispersed and seemed like being damaged (Fig. 2c, pointed with arrows). Further complementation experiment showed that the above cell growth and nucleic defects in the mutant cells were irreversible, inasmuch as the aberrant phenotypes could not be complemented by exogenously expressed ParAB_m_ (i.e. Δ*parAB_m_*/ParAB_m_, a strain in which *parAB_m_* was introduced in trans after the recovery of Δ*parAB_m_*) (Fig. 2a–c). In fact, the Δ*parAB_m_*/ParAB_m_ cells exhibited even more defective phenotypes with respect to growth and nucleoid content and morphology (Fig. 2b and c; Table 2). Together, the above results suggested that in *T. thermophilus*, *parAB_m_* is important for the cell growth and nucleoid morphology maintenance.

**Table 2.** Characteristics of the Δ*parAB_m_* mutant.

Strain	Cell length (μm)	Cell width (μm)	Frequency of anucleate cells	Relative megaplasmid copy number	
				TTP0108 locus	TTP0161 locus
WT	3.93 ± 1.11	0.64 ± 0.05	0.4%	1	1
Δ*parAB_m_*	2.76 ± 1.15	0.83 ± 0.14	8.5%	0.59 ± 0.39	0.61 ± 0.39
Δ*parAB_m_*/ParAB_m_	3.67 ± 1.04	0.78 ± 0.09	9.2%	0.66 ± 0.26	0.63 ± 0.20
*P*	*P* < 0.01	*P* < 0.01	*P* < 0.01	*P* > 0.05	*P* > 0.05

The average cell length and width were calculated from 100 cells of each strain, respectively; for calculation of the frequency of anucleate cells, 500 WT cells, 200 Δ*parAB_m_* cells, and 250 Δ*parAB_m_*/ParAB_m_ cells were counted, respectively. For qPCR, the locus near the chromosomal *oriC* (TTC1609) was chosen as an internal reference amplification region, and the relative quantification 2^−ΔΔCt^ method was used to calculate the relative megaplasmid copy number. The significance of difference of the cell length, cell width, and frequency of anucleate cells between Δ*parAB_m_* (or Δ*parAB_m_*/ParAB_m_) and WT, and the significance of difference of the relative megaplasmid copy number between Δ*parAB_m_* and Δ*parAB_m_*/ParAB_m_ were all tested by Student's *t*-test.

### Genome content analyses of Δ*parAB_m_*

**Fig. 3.**
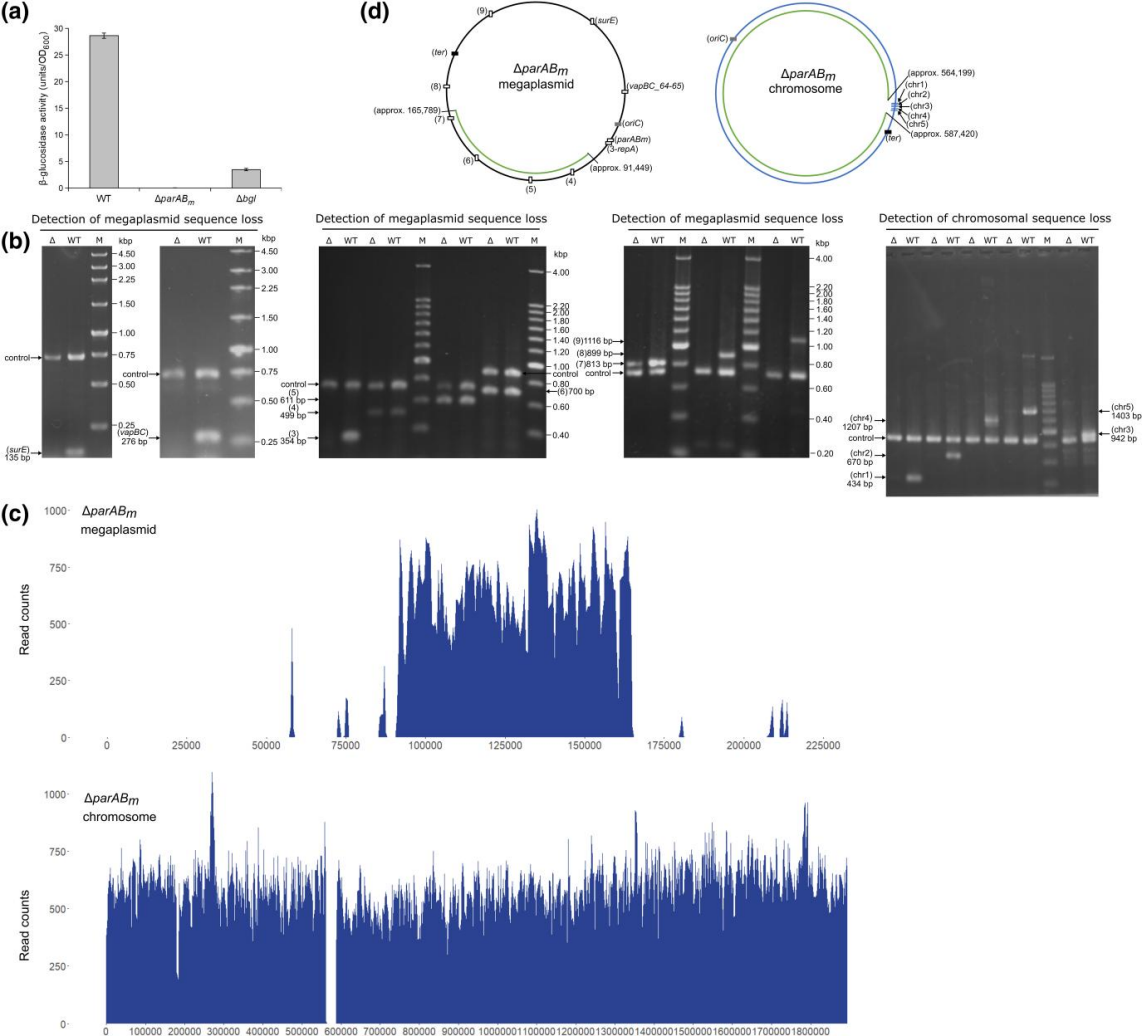
Analyses of the genome content of the Δ*parAB_m_* mutant from various aspects. a) Bgl assay results of WT, Δ*parAB_m_*, and Δ*bgl*. Mean and SD from three biological repeats are shown. b) PCR confirming the megaplasmid and chromosomal sequence loss in Δ*parAB_m_*. In each reaction, two sets of primers were used, which could bind the control gene [TTC0825 (734 bp) or TTC1220 (890 bp) for detecting megaplasmid deletion and TTC0439 (857 bp) for detecting chromosome deletion] and the target amplification region, respectively. The genomic positions of the target amplicons were indicated in d). c) Whole genome sequencing confirming the megaplasmid and chromosomal sequence loss in Δ*parAB_m_*. Shown is the alignment of the Illumina reads to the reference genome of *T. thermophilus* HB27 (NCBI reference sequence: GCA_000008125.1). d) Schematic maps showing the residual megaplasmid and chromosomal regions (inner arcs) in Δ*parAB_m_*. In the megaplasmid map, boxes (i.e. *surE*, *vapBC64_65*, and “1–9”) indicate the 11 genetic loci that have been tested by PCR. In the chromosome map, short lines (i.e. “chr1–5”) indicate the five genetic loci that have been tested by PCR. The origin and terminus regions of the megaplasmid or of the chromosome are shown as *oriC* and *ter*, respectively.

The wild-type *T. thermophilus* cells are orange–yellow due to the ability of producing carotenoid, and the enzyme catalyzing the last step of carotenoid synthesis is encoded by locus TTP0057 on the megaplasmid (Takano *et al*. 2011). Therefore, the white color of the Δ*parAB_m_* colony was an indicative of carotenoid synthesis deficiency (Fig. 2a), probably caused by loss of the TTP0057 locus. To verify this speculation and further uncover the role of *parAB_m_* in genome maintenance, we measured genome content at multiple genomic sites of the mutant via various methods. Enzyme activity assay result showed that Δ*parAB_m_* had nearly no Bgl (encoded by TTP0042 on the megaplasmid) activity (Fig. 3a). The enzyme activity was even lower than the Δ*bgl* mutant (Fig. 3a), indicating that there was not only a complete absence of TTP0042 but also of TTP0222 (encodes β-galactosidase, producing unspecific cleavage of the substrate during enzyme activity test) in the Δ*parAB_m_* mutant. Further, PCR and genome sequencing data (the sequencing data of Δ*parAB_m_* was deposited in the NCBI SRA database under BioProject accession code PRJNA909084) confirmed that in Δ*parAB_m_*, a large portion (∼160 kb) of the megaplasmid and a small portion (∼23 kb) of the chromosome were lost, respectively (Fig. 3b and c). Notably, the same PCR result was observed in all of the three Δ*parAB_m_* mutants (Fig. 3b; Supplementary Fig. 5), implying that the observed genome deletion was probably related to the *parAB_m_* deletion. The coordinates of the eliminated regions were roughly mapped as 1–91,449 and 165,789–232,605 on the megaplasmid and 564,199–587,420 on the chromosome (Fig. 3d). Further investigation of the deletion sites revealed that two 1031-bp direct repeating sequences were, respectively, found at the ends (i.e. 85,677–86,708 and 159,958–160,989) of the truncated megaplasmid region, indicating that the deletion event was probably triggered by homologous recombination. With respect to the chromosome deletion event in Δ*parAB_m_*, we found that there are four insertion sequences (ISs) (i.e. TTC0571 residing in 555,835–556,350; TTC0572 residing in 556,245–556,868; TTC0577 residing in 560,079–561,038; TTC0578 residing in 561,059–562,092) immediately closing to the chromosomal deletion start. The transposases encoded by these ISs might have mediated the deletion. Overall, these observations indicated that *parAB_m_* plays a vital role in genomic sequence (especially megaplasmid sequence) integrity maintenance.

### Chromosome and megaplasmid segregation in Δ*parAB_m_*

**Fig. 4.**
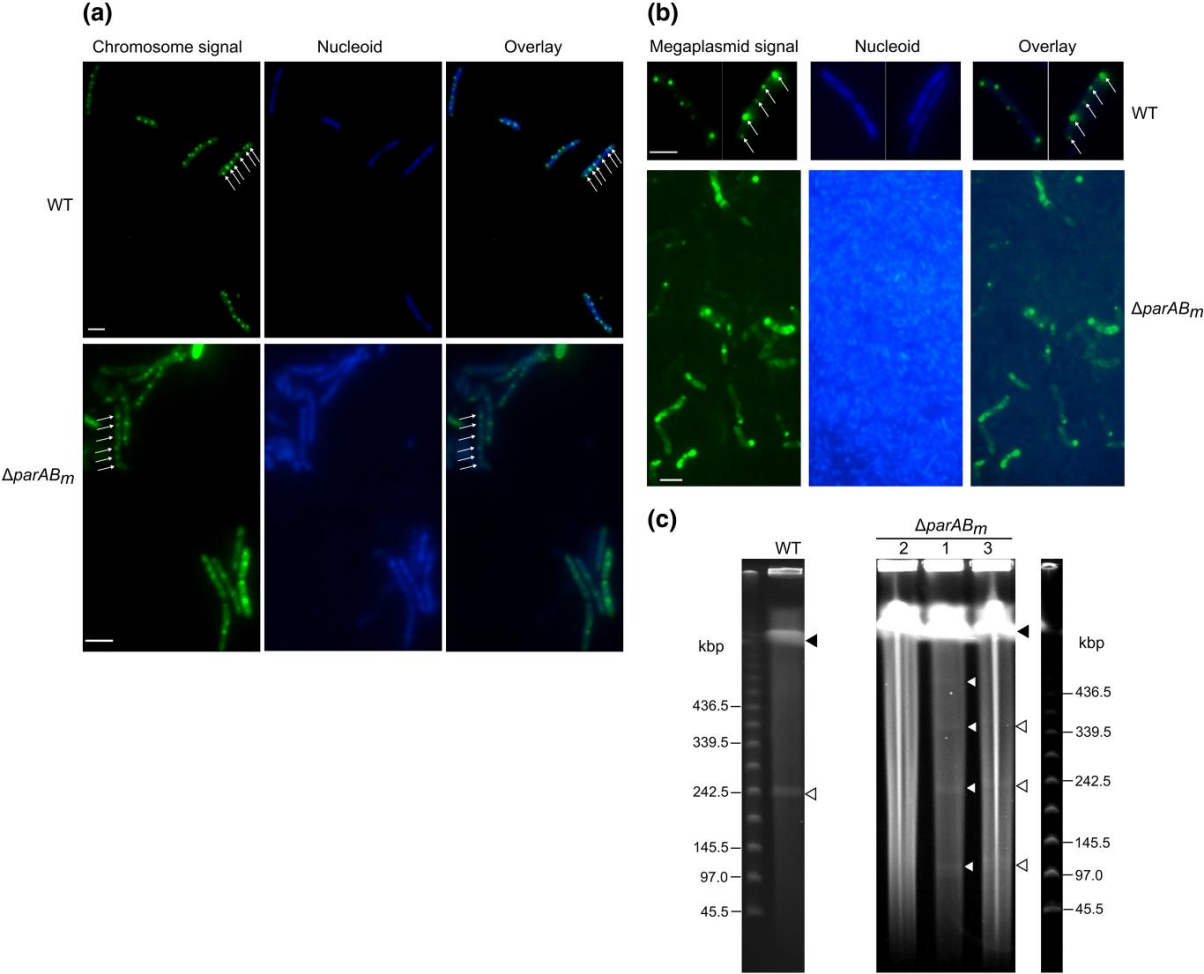
Chromosome and megaplasmid segregation patterns in Δ*parAB_m_*. (a, b) Representative images showing the subcellular localization and segregation patterns of the chromosome (a) and megaplasmid (b) in WT and Δ*parAB_m_*. The chromosomal and megaplasmid signals were represented by ParB_c_-sGFP and ParB_m_-sGFP fluorescent foci, respectively. Arrows are pointing the apparent fluorescent foci. Bars, 2 μm. c) PFGE analysis of the undigested genomic DNA isolated from the WT or Δ*parAB_m_* cells. The arrowheads at the top of the PFGE gel image indicate the chromosome bands, and the other arrowheads indicate the megaplasmid bands. 1, 2, and 3 indicate the PFGE result of the three Δ*parAB_m_* mutants. In mutant 2, the genomic DNA was completely degraded, and although with genome degradation, mutants 1 and 3 showed faint ladder-like megaplasmid bands. Note: in both WT and mutant, the circular megaplasmids ran slower than the corresponding linear marker molecule during PFGE.

qPCR results showed that the average copy number of the truncated megaplasmid in the *parAB_m_* mutant was lower than that of the WT strain (Table 2). This result implied that the residual megaplasmid in the Δ*parAB_m_* cells was frequently lost during cell division. To visualize the subcellular localization and segregation patterns of the chromosome and residual megaplasmid, the ParB-*parS* fluorescent tagging system was utilized (see *Materials and methods*). ParB_c_-sGFP (from the chromosomally encoded *parABS_c_* system) and ParB_m_-sGFP would specifically bind their cognate *cis*-acting *parS* sequence forming fluorescent foci; thus, their localization pattern reflects that of the chromosome and megaplasmid, respectively. Nearly 95 and 54% WT cells contained detectable ParB_c_-sGFP/*parS_c_* and ParB_m_-sGFP/*parS_m_* foci, respectively (Fig. 4a and b), and the average foci numbers were 6.84 ± 2.15 and 4.50 ± 1.64, respectively (100 cells from different microscopic fields were counted). Ninety-three percent of Δ*parAB_m_* cells contained ParB_c_-sGFP/*parS_c_* foci (the average number was 5.31 ± 1.53, 100 cells from different microscopic fields were counted), and the localization pattern of these foci was similar to that of the WT foci (Fig. 4a). However, extremely few Δ*parAB_m_* cells (lower than 10%) were found possessing the ParB_m_-sGFP/*parS_m_* signal (Fig. 4b), suggesting that a fraction of the mutant cells had lost the residual megaplasmid, which was in high agreement with the qPCR result. Further, the ParB_m_-sGFP/*parS_m_* fluorescent foci in the WT cells were regularly spaced and orderly arranged; on the contrary, in the Δ*parAB_m_* cells (especially irregularly shaped cells), they were aggregated and randomly distributed throughout the cell (Fig. 4b). Moreover, PFGE analysis also showed that doubled or multiplied megaplasmid sizes could be detected in Δ*parAB_m_* (Fig. 4c). Together, these results indicated that in the *parAB_m_* deletion cells, the chromosomes could be normally segregated; however, the replicated residual megaplasmid copies could not be properly separated or localized to the correct cellular position, thus forming dimer or multimer.

### Viability of the Δ*parAB_m_* cells that have lost megaplasmid

**Fig. 5.**
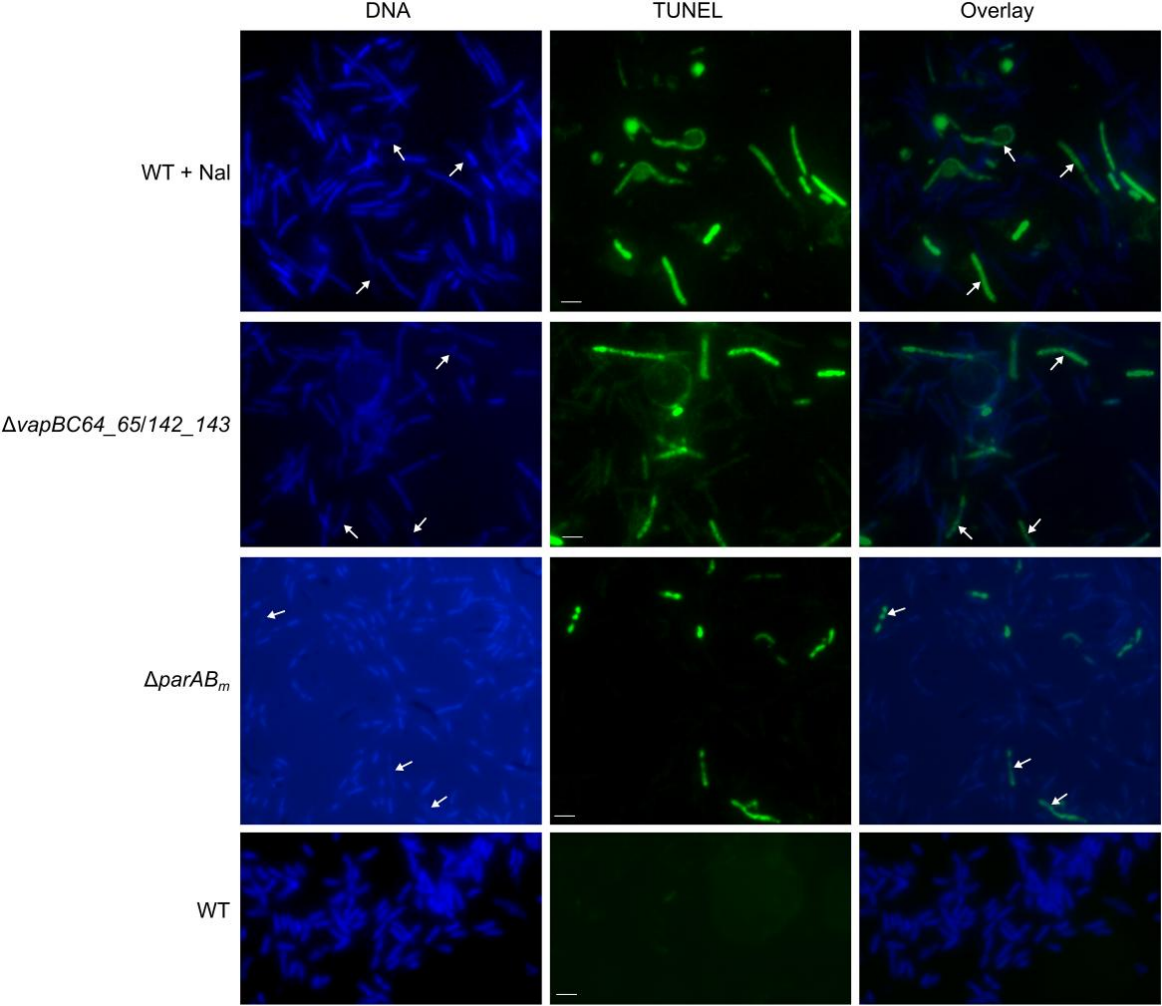
TUNEL assay of the Δ*parAB_m_* cells, Δ*vapBC64_65*/*142_143* cells, and Nal-treated WT cells. Apparent broken nucleoids are pointed by arrows. Bars, 2 μm.

The nucleoids of certain Δ*parAB_m_* cells especially abnormal cells were dispersed or broken (Fig. 2c), implying nucleoid fragmentation and cell death of these cells. The PFGE result also indicated that the nucleoids were severely degraded in the mutant (this phenomenon was reproducible in all of the three generated *parAB_m_* mutants) (Fig. 4c). The occurrence of the DNA broken event in the Δ*parAB_m_* cells was further analyzed by TUNEL assay. For TUNEL, Nal- (be able to cause nucleoid fragmentation) treated WT cells were used as positive controls. Around 67% of irregularly shaped Δ*parAB_m_* cells with nucleoids dispersed (100 aberrant cells from different microscopic fields were counted) showed positive TUNEL fluorescence signal similar to that of the Nal-treated cells (Fig. 5), indicating that DNA degradation had occurred in these cells, which could trigger cell death.

The *T. thermophilus* megaplasmid encodes two sets of TA modules belonging to the VapBC family, i.e. TTP0064/TTP0065 and TTP0142/TTP0143. In WT cells, the cognate antitoxins can probably counteract the activity of toxins; however, when both TA encoding genes were lost, toxins would exert damage effect, since toxins are more stable than antitoxins. In Δ*parAB_m_*, the TTP0064/TTP0065 TA locus was lost (as shown in Fig. 3d), and in some cells, the residual megaplasmid was completely cured (Fig. 4b); thus, the second TA locus (TTP0142/TTP0143) was also lost in these cells. It is possible that the observed DNA fragmentation and cell death phenomenon in Δ*parAB_m_* were caused by the activity of the two toxins. To test this, we deleted both TA loci and performed TUNEL assay of the Δ*vapBC64_65*/*142_143* mutant (Supplementary Fig. 6). Although the average cell length of the *vapBC* mutant was much longer than that of Δ*parAB_m_*, the nucleoid morphology of the majority of the Δ*vapBC64_65*/*142_143* cells was resembling to that of the Δ*parAB_m_* cells, which appeared as broken punctates (Fig. 5). Further, around 18% of the Δ*vapBC64_65*/*142_143* cells (100 cells from different microscopic fields were counted) had similar TUNEL fluorescence as the aberrant Δ*parAB_m_* cells and Nal-treated cells. Overall, the above results suggested that deletion of *parAB_m_* could eventually trigger loss of the whole megaplasmid in certain cells, and thus loss of the megaplasmid-encoded TA systems, which was threatening to the viability of these *T. thermophilus* cells.

## Discussion

Compared with those of the chromosomal and low-copy-number plasmid-encoded *parABS* systems, the characteristics of the megaplasmid-encoded *parABS* ortholog were barely understood. Further, whether the *parABS* system can function to partition genomes in polyploid bacteria was also unknown. *T. thermophilus* contains multiple copies of megaplasmid which encodes *parABS*; therefore, it is an ideal organism for solving the above queries.

### The *parABS_m_* system works to guard the genome sequences

To delete the *parAB_m_* operon, we co-transformed a *parAB_m_* gene deletion vector (pUC-Δ*parAB_m_::blm*) and an exogenous plasmid encoding ParAB_m_ (pMK-*parAB_m_*) to the WT *T. thermophilus* cells. During the transformation, the cells were grown for 3 h after addition of the plasmid DNAs. In the beginning, the megaplasmid *parAB_m_* (including *parS_m_* on *parB_m_*) was present in the cells, and the megaplasmid/plasmid-expressed ParB_m_ would bind the *parS_m_* site; thus, the whole ParA_m_–ParB_m_–*parS_m_* system would work properly. However, the ParA_m_ and ParB_m_ proteins were constantly overexpressed from pMK-*parAB_m_*, thus after a period of time, they would eventually render the megaplasmid-encoded ParAB_m_ unessential. Sequentially, the *blm* antibiotic marker from pUC-Δ*parAB_m_::blm* would easily replace the *parAB_m_* operon (as illustrated in Supplementary Fig. 2), thereby resulting the complete *parAB_m_* deletion.

Through deletion of the *parAB_m_* operon followed by phenotype observation, we found that the *T. thermophilus parABS_m_* system was not only important for the megaplasmid segregation but surprisingly also played a role in genome integrity maintenance. PCR, genome sequencing, and PFGE experiments showed that the Δ*parAB_m_* mutant had lost ∼160 kb (including the *oriC* region and the megaplasmid replication initiator gene *repA*) of the megaplasmid and ∼23 kb of the chromosome sequences, respectively (Fig. 3b–d; Fig. 4c). Notably, there are two long direct repeats (1031 bp) at the two ends of the lost megaplasmid region and four ISs adjacent to the chromosomal deletion start. Therefore, it is highly possible that the megaplasmid deletion was caused by homologous recombination between the two repeats, and the chromosomal deletion might be mediated by transposition of these ISs. Regarding how the residual megaplasmid replicated after loss of the *oriC* site, we hypothesized that it would either integrate into the chromosome or maintain auto-replicating by itself. According to the PFGE data (Fig. 4c), we found that the residual megaplasmid was not integrated into the chromosome (if it was, there should be no megaplasmid bands upon PFGE analysis). Thus, it is possible that the megaplasmid would choose another site as the *oriC* site, and the replication initiation protein might be from the chromosome. Indeed, unlike that of the monoploid bacteria, the GC skew (software for detecting bacterial replication origin and terminus regions) (Grigoriev 1998) result of the *T. thermophilus* megaplasmid is asymmetrical and contains many high-G/high-C shift points (Supplementary Fig. 7). A similar GC skew pattern was also observed in the genomes of other polyploid bacteria, such as *Synechococcus elongatus* PCC 7942, *Synechocystis* sp. PCC 6803, *Gloeobacter violaceus* PCC 7421, and *D. radiodurans* (Watanabe 2020). This indicated that the megaplasmid of *T. thermophilus* contains multiple *oriC*-like regions, which may support autonomous replication after the most qualified *oriC* was deleted. A same result has been uncovered in *Anabaena* sp. PCC 7120 (Watanabe 2020). Further, as demonstrated by Fig. 2, the phenotypic defects (e.g. the cell growth, morphological and nucleic defects) of the Δ*parAB_m_* mutant could not be complemented by the exogenously expressed ParAB_m_. This result was as expected. Deletion of the megaplasmid *parAB_m_* in Δ*parAB_m_* meant loss of the megaplasmid *parS_m_* site (residing in *parB_m_*); therefore, there was no *cis*-reacting site of the ParB_m_ protein during the complementation experiment. Additionally, the mutant cell has already lost a large portion of the genome sequence. Nevertheless, it is worthy to note that this result does not contradict the principle that we used to generate the Δ*parAB_m_* mutant, due to the fact that during the mutant generation process, *parS_m_* was present at first and was lost afterward (see *Materials and methods*).

Moreover, we found that when *parAB_m_* was deleted, the replicated megaplasmid could not be transmitted to the correct cellular position or segregated properly (Fig. 4b and c), thus forming intertwined structure in which the repeating regions might directly contact each other, whereby recombinases might easily recognize the complex and execute sequence excision. This interpretation is reasonable, as although plasmids (especially multicopy number plasmids) can provide certain benefits for bacteria, they are meanwhile metabolic burdens to the cell (Million-Weaver and Camps 2014). To increase their stability, plasmids usually carry dedicated genetic determinants, namely, plasmid partitioning determinants (e.g. the *parABS* system) (Bouet and Funnell 2019; Baxter *et al*. 2020), post-segregational killing systems (e.g. the TA system) (Hernández-Arriaga *et al*. 2014), and multimer resolution systems (some recombinases, e.g. XerCD) (Colloms 2013; Cameranesi *et al*. 2018). In addition to these three plasmid stability elements, ISs are also suggested to be important for the architecture of plasmids (or even chromosomes) (Bardaji *et al*. 2011). When bacteria were subjected to growth stress or when the plasmid stability determinants were missing, genetic changes of the plasmids could possibly occur (Bardaji *et al*. 2019; Ramijan *et al*. 2020). Loss or rearrangement of plasmid sequences was also observed in some other bacteria. For example, genomes of actinobacteria were shown to readily undergo rearrangements or deletions, and the size of the deletion genome could be more than 1 Mb (Hoff *et al*. 2018). Ramijan *et al*. (2020) provided evidence showing that protoplast formation and regeneration (i.e. a stressful process) in the filamentous actinobacterium *Kitasatospora viridifaciens* could trigger severe megaplasmid sequence loss and genome rearrangement. In *Pseudomonas syringae*, inactivation of the TA systems from its virulence plasmid pPsv48C could also lead to high-frequent deletions of the plasmid sequence (Bardaji *et al*. 2019). In the case of *K. viridifaciens*, the genome variation was suggested to be the consequence of transpositions of IS upon environmental stress (Ramijan *et al*. 2020). In *P. syringae*, that was triggered by recombinations between two copies of miniature inverted-repeat transposable elements (MITEs) or one-ended transposition of IS*801* once the TA system was lost (Bardaji *et al*. 2019). The similar event has also been revealed in *Shigella sonnei*, in which the absence of plasmid TA systems (i.e. *ccdAB* and *gmvAT*) was found to contribute to pINV loss, and organization of ISs on pINV could determine plasmid plasticity (Martyn *et al*. 2022).

In our situation, it seemed that the large genome sequence deletion in Δ*parAB_m_* was fundamentally caused by the deletion of the *parAB_m_* genes; since the genome deletion at the same region was not a random event, it was found in all of the three Δ*parAB_m_* mutants (Fig. 3b; Supplementary Fig. 5). Unlike the situations in the bacteria exemplified above, it was not caused by the loss of the TA system, on account of that genome deletion at the same region as that of Δ*parAB_m_* was not found in the Δ*vapBC64_65*/*142_143* mutant (the sequencing data was deposited in the NCBI SRA database under accession code PRJNA909084) (Supplementary Figs. 8 and 9). Further, it seemed that the large genome deletion did also not happen before the *parAB_m_* deletion, since there were no important or potential genes in the deleted genomic region of Δ*parAB_m_* that had to be lost to allow for the survival of the *parAB_m_* mutant (Supplementary Table 2). In fact, without the initial generation of the Δ*parAB_m_*::*blm* genotype, a spontaneous recombination of the two repeating sequences (1031 bp) of the megaplasmid would not possibly happen. The two repeats are genomically and also spatially separated (Henne *et al*. 2004), and in the numerous previous studies from our and other groups (Ohtani *et al*. 2010, 2016; Angelov *et al*. 2013; Li *et al*. 2015; Li 2019b), no spontaneous recombination event of the two repeating elements could be observed in the WT strain or in any other megaplasmid gene deletion mutants (typically, also not observed in the *vapBC* deletion mutant in this study). Apparently, when *parAB_m_* was deleted, the megaplasmid could not be segregated properly (Fig. 4a and b), thus became entangled (Fig. 4b and c); at this moment, the repeating elements might directly interact each other, and thereby the recombinases would recognize the complex and perform the excision. Taken together, it seems logical that the large megaplasmid sequence (including the later inserted *blm* marker) in Δ*parAB_m_* was lost by homologous recombination, after the *parAB_m_* genes were deleted.

Regarding the mechanisms for how loss of TA could lead to deletion of genome sequences, it has been previously shown that in *Shigella* spp., the VapBC family TA system could stabilize local sequences by preventing IS-mediated deletions (Pilla *et al*. 2017). Therefore, we hypothesized that the megaplasmid *parAB_m_* might work to guard the genome sequence in a similar mechanism. Intriguingly, to our knowledge, before this study, none of the plasmid or chromosome deletion event was found to be directly attributed to *parAB* disruption, although it is theoretically highly possible. The reason could be that for the low-copy-number plasmid, the *parABS* system is primarily important, disruption of which would cause partitioning defect, thus loss of the whole plasmid but not a portion of sequence (Million-Weaver and Camps 2014; Baxter and Funnell 2014; Bouet and Funnell 2019). For the chromosomes, the *parABS* system is not significant for its maintenance, as redundant mechanisms (e.g. SMC, TopoIV, and Ftsk-XerCD systems) can act to segregate the chromosome (Badrinarayanan *et al*. 2015). Numerous studies have shown that deletion of *parABS* had negligible effect for the chromosome segregation (Lewis *et al*. 2002; Bartosik *et al*. 2004; Fogel and Waldor 2006; Lasocki *et al*. 2007; Li *et al*. 2015). To some extent, the *T. thermophilus* megaplasmid can be defined as a second chromosome, as it carries certain essential genes for cell viability (e.g. TTP0161 and TTP0162 encoding the α and β subunit of class I ribonucleotide reductase which is a key enzyme for deoxyribonucleotide synthesis) (Henne *et al*. 2004; Ohtani *et al*. 2016). However, on the other hand, it is still a metabolic burden for the whole cell due to its big genome size. Therefore, it is conceivable that the *parABS_m_* system might have been evolved to protect the essential sequences but not the whole megaplasmid.

### The *parABS_m_* system can determine cell viability

In addition to causing genome sequence deletion, the TA systems can also determine cell viability (Kamruzzaman *et al*. 2021). The cells with TA loci lost might be aberrantly shaped, undivided, or have fragmented nucleoid. For example, when the megaplasmid-derived chromosome (ChrII) of *V. cholerae* was eliminated upon *parAB2* deletion, the 13 TA loci on which were also eliminated, such that the toxins started to cause detrimental cytologic changes. Consequently, the cells became hypertrophic, undivided, and had condensed nucleoid, which would die eventually (Yamaichi *et al*. 2007). A number of TA deletion mutants have been shown to have distinct or deleterious phenotypes (Arcus *et al*. 2011). For example, Δ*fitAB* mutant of *Neisseria gonorrhoeae* showed a faster traversing rate through epithelial cells than the wild type (Wilbur *et al*. 2005); a *hipBA* deletion mutant of *E. coli* showed a reduction in antibiotic tolerance (Schumacher *et al*. 2009); and deletion of *vapBC3* and *vapBC4* could impair the infection ability of *Mycobacterium tuberculosis* in animal models (Agarwal *et al*. 2018). It has been suggested that besides its ability of degrading RNA or glutamyl-tRNA synthetase (Christensen and Gerdes 2003; Germain *et al*. 2013), the TA systems (e.g. VapBC) can also regulate the intracellular metabolisms, such as to regulate the levels of branched-chain amino acids which are proposed to play essential roles in monitoring the nutritional supply and physiological state of the cell (Frampton *et al*. 2012). For instance, deletions of the total three TA loci (VapBC, MazEF, and Phd/Doc, ΔTAtriple) in *Mycobacterium smegmatis* could result in severe survival defect. Further experiments showed that there was a significant difference in the levels of branched-chain amino acids between the wild-type and the ΔTAtriple mutant, which would cause DNA damage and cell death. We thus hypothesized that the megaplasmid-encoded TA loci of *T. thermophilus* may also play roles in the intracellular metabolism regulations. Under this circumstance, when both the toxin and antitoxin were absent, the ongoing phenotypes of the Δ*vapBC64_65*/*142_143* mutant should be possibly observed (Fig. 5). In the future work, it is worthy to test the metabolic changes of this mutant. Nevertheless, in our study, deletion of *parAB_m_* led to the excision of ∼160 kb megaplasmid sequences, which included one TA locus (*vapBC64_65*). The smaller megaplasmid would not be segregated correctly; thereby, certain daughter cells eventually lost the whole megaplasmid. These cells became swell or twisted and had dispersed nucleoids (Fig. 2c). TUNEL assay showed that they were actually dead cells (Fig. 5). The nucleoid morphology of these cells was similar to that of the Δ*vapBC64_65*/*142_143* mutant (Fig. 5). Therefore, we suggested that the combined actions of the megaplasmid-encoded toxins likely to some aspects contributed to the observed phenotype of the dead Δ*parAB_m_* cells. This indicated that through guarding the genome sequences (including the TA loci), the *T. thermophilus parABS_m_* system can also determine cell viability.

### The *parABS_m_* system mediates the megaplasmid segregation

Besides its role in genome integrity maintenance, *parABS_m_* was also involved in megaplasmid localization and segregation. Fluorescent tracking of the Δ*parAB_m_* genomes demonstrated that the truncated megaplasmid was frequently mislocalized or even completely disappeared (Fig. 4b). Also, the PFGE analysis showed that the smaller megaplasmid was likely to form multimers (Fig. 4c). These results indicated that although the megaplasmid present with multicopy in one *T. thermophilus* cell, it still requires active segregation machineries. High-copy-number plasmids (>20) normally lack active partition systems and are believed to be segregated randomly during cell division, since the frequency of daughter cells receiving no plasmid is practically low (Million-Weaver and Camps 2014). Our data suggested that in *T. thermophilus*, the copy number of the megaplasmid was not sufficient for random segregation event to occur. Additionally, the megaplasmid copies were regularly arranged and equally spaced in the WT cells (Fig. 4b), which also implied that they could be stringently segregated. Further, it seemed that except for *parABS_m_*, there was no redundancy of the mechanisms that account for the megaplasmid segregation in *T. thermophilus*. This was in a striking contrast to the segregation of chromosomes but analogous to that of the low-copy-number plasmids. For most of the bacterial chromosomes, deletions of the *parAB* genes, their segregations would proceed normally (Lewis *et al*. 2002; Bartosik *et al*. 2004; Fogel and Waldor 2006; Lasocki *et al*. 2007; Li *et al*. 2015). For instance, deletion of the *parA1* gene in *V. cholerae* did not cause evident chromosome segregation defect (Fogel and Waldor 2006), and in the *B. subtilis parB* (*spo0J*) deletion mutant, most cells still exhibited regular chromosome segregations (Lee and Grossman 2006). In fact, it is now widely accepted that the chromosomal *parABS* system mainly functions to motivate the chromosomal origin region but not bulk DNA segregation (Jalal and Le 2020). The segregation of the bulk chromosomal DNA is accomplished by SMC complex (structural maintenance of chromosomes) and in the meanwhile disentangled by topoisomerase (e.g. TopoIV) (Badrinarayanan *et al*. 2015). Moreover, the *T. thermophilus* megaplasmid *parABS_m_* system is phylogenetically more close to the plasmid-encoded *parABS* (Li *et al*. 2015). Together, our results indicated that unlike the chromosomal *parABS*, the megaplasmid *parABS* system was important for mediating the megaplasmid segregation, even under the context that the replicon was present with multicopy in one cell.

### Conclusions

Through providing a plasmid-born copy of *parAB_m_*, we generated the *T. thermophilus parAB_m_* gene deletion mutant. The genome content analysis results showed that the Δ*parAB_m_* mutant had lost ∼160 kb of the megaplasmid and ∼23 kb of the chromosomal sequences, respectively. The truncated megaplasmid would not be segregated correctly; thus, certain daughter cells eventually lost the entire megaplasmid and became twisted or enlarged containing dispersed nucleoids. We further found that when the megaplasmid was lost, the megaplasmid-encoded TA systems (VapBC64_65 and VapBC142_143) were also eliminated; thereby, the toxins would exert detrimental effects, such as to fragment DNA. Since the genome sequence deletion at the same region was not detected in the Δ*vapBC64_65*/*142_143* mutant, we concluded that the megaplasmid and chromosome excision event in Δ*parAB_m_* was related to the deletion of *parAB_m_*. Thus, it seems that ParAB_m_ can act to prevent recombinase or transposase-mediated genome instability. To sum up, our results suggested that in *T. thermophilus*, the megaplasmid *parABS* system plays essential roles in both megaplasmid segregation and genome integrity maintenance. We initially characterized the functions of a megaplasmid-encoded *parABS* system in a polyploid thermophilic bacterium and revealed a new factor (i.e. *parABS*) for determining bacterial genome integrity.

## Supplementary Material

jkad038_Supplementary_Data

## Data Availability

Strains and plasmids are available upon request. The authors affirm that all data necessary for confirming the conclusions of the article are present within the article, figures, and supplemental material. Supplementary file contains all supplemental tables and figures. The sequencing data was deposited in the NCBI SRA database under accession code PRJNA909084. Supplemental material available at G3 online.
