# Supplementary material for: The parABSm system is involved in megaplasmid partitioning and genome integrity maintenance in Thermus thermophilus
Source: G3 (Bethesda). 2023 Feb 14;13(4):jkad038. doi: 10.1093/g3journal/jkad038 (PMC10085754; doi:10.1093/g3journal/jkad038)
Supplement: jkad038_Supplementary_Data [file jkad038_supplementary_data.docx]

**The *parABS_m_* system is involved in megaplasmid partitioning and genome integrity maintenance in *Thermus thermophilus***

Haijuan Li^1*^, Lingling Xu^1^, Xiaoxiao Li^1^

^1^College of Biological and Environmental Engineering, Xi'an University, No. 168 South Taibai Road, 710065 Xi'an, China

*****Correspondence: [haijuanli@xawl.edu.cn](mailto:haijuanli@xawl.edu.cn)

**Supplementary materials**

**Supplementary Table 1. Primers used in this study**

| **Name** | **Sequences (5’-3’)^*^** | **Usage** |
| --- | --- | --- |
| parAB_m_F1-F | tgcatgcctgcaggtcgactAACATCCACGGTGCCGTAGG | amplifying *parAB_m_* downstream ﬂanking  region for pUC-Δ*parAB_m_::blm* |
| parAB_m_F1-R | gactgatctagaggatcccccGCCCTCTCGTTTGTTTCTC |  |
| parAB_m_F2-F | gtttctgttatactcccgggGGGCCTAGACTATCCCAAG | amplifying *parAB_m_* upstream ﬂanking  region for pUC-Δ*parAB_m_::blm* |
| parAB_m_F2-R | gctcggtacccggggatcctctagACGAGACCGGGAAGTACGAG |  |
| blm-F | GGGGATCCTCTAGATCAGTC | amplifying *blm* for pUC-Δ*parAB_m_::blm* and pUC-Δ*vapBC142_143::blm* |
| blm-R | CCCGGGAGTATAACAGAAAC |  |
| dparA_m_-F | TTCTCCAGCACCCGGTTGTC | PCR confirmation of *parA_m_* gene deletion in Δ*parAB_m_* |
| dparA_m_-R | CTACTGGTACGTGCGGGAAC |  |
| dparB_m_-F | GCCTTCTCCTTCCGCAAGAC | PCR confirmation of *parB_m_* gene deletion in Δ*parAB_m_* |
| dparB_m_-R | TCCTGGACCTCTCCGAGAAG |  |
| dparAB_m_-F | AGTCAAGGCCACGGGTGTCTTC | PCR confirmation of the heterozygous Δ*parAB_m_::blm* mutant |
| dparAB_m_-R | CCAGACCATCGTCTACGTCTTC |  |
| TTC0213-F | TCCTGATCCTCACGGGAAGC | amplifying the control region (TTC0213) when confirming *parAB_m_* deletion in Δ*parAB_m_* |
| TTC0213-R | TCGGGGGCGTCCTCATCGGC |  |
| parB_c_-F1-F | tgcatgcctgcaggtcgactTCGGCTTCCTCAAGCTCTTC | amplifying downstream ﬂanking region for  pUC::Δ*parB_c_::kat-parB_c__sgfp* |
| parB_c_-F1-R | gacgagctctacaagtaagaCTACCAGGCGTAGCCGAAGA |  |
| parB_c_-F2-F | gtcaagcggtcgggccttaaCTAGGCTTCCTGGACACGGG | amplifying upstream ﬂanking region for  pUC::Δ*parB_c_::kat-parB_c__sgfp* |
| parB_c_-F2-R | gctcggtacccggggatcctGCCTTCCCTTGAAGATCGTC |  |
| parB_c_-gfp-F | TTAAGGCCCGACCGCTTGAC | amplifying *kat-parB_c__sgfp* for pUC::Δ*parB_c_::kat-parB_c__sgfp* |
| parB_c_-gfp-R | TCTTACTTGTAGAGCTCGTC |  |
| fdh-F1-F | tgcatgcctgcaggtcgactTTAGGGCCATCTCCCTTCGG | amplifying downstream ﬂanking region for  pUC::Δ*fdh::kat-parB_m__sgfp* |
| fdh-F1-R | gtcaagcggtcgggccttaaACATCCCGGCCTACAAGGAG |  |
| fdh-F2-F | gacgagctctacaagtaagaTTCTCCGGGTTCACGGGAAG | amplifying upstream ﬂanking region for  pUC::Δ*fdh::kat-parB_m__sgfp* |
| fdh-F2-R | gctcggtacccggggatcctTTGGGAGAACCGGGACAAC |  |
| parBm-gfp-F | TTAAGGCCCGACCGCTTGAC | amplifying *kat-parB_m__sgfp* for pUC::Δ*fdh::kat-parB_m__sgfp* |
| parBm-gfp-R | TCTTACTTGTAGAGCTCGTC |  |
| vapBC_6465_F1-F | tgcatgcctgcaggtcgactTTCCGCTCCCAAAGGTACCC | amplifying upstream ﬂanking region for  pUC-Δ*vapBC64_65::kat*; the forward primer was also used for determining Δ*vapBC64_65::kat* |
| vapBC_6465_F1-R | gggatacttggcaaacgccaTGATGGGCAAGCGAGGCTTC |  |
| vapBC_6465_F2-F | gtttctgttatactcccgggCGAGGAGTTCCGCCACGTTC | amplifying downstream ﬂanking region for  pUC-Δ*vapBC64_65::kat*; the reverse primer was also used for determining Δ*vapBC64_65::kat* |
| vapBC_6465_F2-R | gctcggtacccggggatcctAAGTGGATCATGGCGTAGAG |  |
| kat-F | TGGCGTTTGCCAAGTATCCC | amplifying *kat* for  pUC-Δ*vapBC64_65::kat* |
| kat-R | CCCGGGAGTATAACAGAAAC |  |
| vapBC_142-3_F1-F | tgcatgcctgcaggtcgactCTCAACTGGGCCGAGCTTTC | amplifying upstream ﬂanking region for  pUC-Δ*vapBC142_143::blm*; the forward primer was also used for determining Δ*vapBC64_65/142_143* |
| vapBC_142-3_F1-R | gactgatctagaggatccccTCAATCCCTGGGAGGAATAG |  |
| vapBC_142-3_F2-F | gtttctgttatactcccgggGCTTTGGCTTCTTGGAGTTG | amplifying downstream ﬂanking region for  pUC-Δ*vapBC142_143::blm*; the reverse primer was also used for determining Δ*vapBC64_65/142_143* |
| vapBC_142-3_F2-R | cggtacccggggatcctAGTACCGTATGCGGGTTGAC |  |
| pMK18-F | GGATGTGCTGCAAGGCGATTAAGTTGG | amplifying pMK18 backbone for pMK18-*parAB_m_* |
| pMK18-R | TCAAAATGGTATGCGTTTTG |  |
| parAB_m_-F | caaaacgcataccattttgaTGGGGGATACTTGGCAAACG | amplifying *parAB_m_* for pMK18-*parAB_m_* |
| parAB_m_-R | aatcgccttgcagcacacatccAACGAGAGGGCGTTACCTCG |  |
| TTP0180-F | GCCACTTGACACGACGGAAC | qPCR for measuring megaplasmid copy number (TTP0180 locus) |
| TTP0180-R | CTCGCTGAAACGGTGTACCC |  |
| TTP0161-F | ACGGAGTTCAGCTTCCAGTC | qPCR for measuring megaplasmid copy number (TTP0161 locus) |
| TTP0161-R | ATCTCGGGCTTAGGAAAGCG |  |
| TTC1609-F | CTCCTCTCGGACCAGCTTTC | qPCR for measuring chromosome copy number (TTC1609 near the *oriC* region) |
| TTC1609-R | TGGTCCCGAAGAGGATCAAG |  |
| surE-F | TCGAGGCGAACCAGTAGAAG | the primer pairs for detecting the megaplasmid sequence loss in Δ*parAB_m_* or Δ*vapBC64_65/142_143* |
| surE-R | CCTTCCTGGTGAACGTGAAC |  |
| vap-F | TGCTGGACTGGGAGACCTAC |  |
| vap-R | ACCAGTTCGCCCAAGGTGAG |  |
| 3-F | GCTCCTCTACCACCTGTCTG |  |
| 3-R | ACATCAAGGAGCGGCTGGAG |  |
| 4-F | CCTCCTGTGGCTTTCTATC |  |
| 4-R | GCTCTGGAGAGGAGTTTG |  |
| 5-F | CACCATCCAGCGCAGAAAGC |  |
| 5-R | ACGACTTCCGGCCCGATTAC |  |
| 6-F | AGGCGTAGGGAAGGTTGTC |  |
| 6-R | AGGCGTAGATGGGAGGAAG |  |
| 7-F | CAGGACGCCCAAGACTTAG |  |
| 7-R | CGGACTGGAAGCTGAACTC |  |
| 8-F | CCGCACAGTATCTCGGTCTC |  |
| 8-R | CAGGAAGCGCCCTCTTAAGC |  |
| 9-F | CCTACCTCGCCTTCTTCTAC |  |
| 9-R | CCACCAGGAAGCGGAAGGCC |  |
| 10-F | TCCTGGTCCAGTGAAGACAAG |  |
| 10-R | TGGCGTTGGACCTGATGGAA |  |
| 11-F | TGGACACCGATCAGGTAAC |  |
| 11-R | GGTGGGTATGCGATTCAAG |  |
| 12-F | CGCCTGGAGAACGTCTTGTG |  |
| 12-R | GCTCTTTCGCCGACAACGTG |  |
| 13-F | CTTCGGCCTGTGGAACTTCG |  |
| 13-R | GTCATAGGCGTTTCTCCTCC |  |
| 14-F | GGGAGCAGGTGTACTAAAGC |  |
| 14-R | TTCGCCGTGGTGGACGTTTG |  |
| chr1-F | GAAAGGGCCTTGCGGATCTC | the primer pairs for detecting the chromosome sequence loss in Δ*parAB_m_* and Δ*vapBC64_65*/*142_143* |
| chr1-R | AAGGAGGTGCTGGCCGAAAC |  |
| chr2-F | GCCTGGAGCTCAAGGGAAAG |  |
| chr2-R | TCAAACTGGCCCAGGAAACG |  |
| chr3-F | GGGTGGCGCTTTCCATGTAG |  |
| chr3-R | TCAAGCGGGAAAGCCTGGAG |  |
| chr4-F | CCGTGGTCTTCCAGATCTTC |  |
| chr4-R | CCCTTCTCCGTCTGCTTCAC |  |
| chr5-F | GTTCCTCTACGCCCACTTCC |  |
| chr5-R | AGGTTGTCCTCCACGGATAG |  |
| TTC0825-F | ACCATGCACCACAAGGTCAT | amplified the control region (TTC0825) when determined megaplasmid deletion in Δ*parAB_m_* and Δ*vapBC64_65*/*142_143* |
| TTC0825-R | GGAGGAGGTGCTCCCTCACG |  |
| TTC1220-F | GCATCGACTACGAGACCCCC | amplified the control region (TTC1220) when determined megaplasmid deletion in Δ*parAB_m_* |
| TTC1220-R | GGCCCTTGAGCACCCGAATG |  |
| TTC0439-F | CTTCCCGCGGCGAAGAGGAC | amplifying the control region (TTC0439) when determined chromosome deletion in Δ*parAB_m_* and Δ*vapBC64_65/142_143* |
| TTC0439-R | AAGATGGCGAGGTCCTTCAG |  |

^*^Lowercases indicate primer sequences for generating overlaps between inserting fragments, or between inserts and vectors

**Supplementary Table 2. The lost genes in the deleted chromosomal region of the Δ*parAB_m_* mutant**

| **Position in the chromosome** | **Locus name** | **Gene product** | **Potential functions** |
| --- | --- | --- | --- |
| 564035-564787 | TTC0581 | triose-phosphate isomerase | probably involved in glycolytic pathway |
| 564796-565833 | TTC0582 | fatty acid desaturase | involved in fatty acid desaturation |
| 565907-566365 | TTC0583 | D-aminoacyl-tRNA deacylase | deacylation of D-aminoacyl-tRNA |
| 566369-567220 | TTC0584 | probable degV protein (EDD domain protein, DegV family) | probably involved in activation of fatty acids |
| 567195-568037 | TTC0585 | DegV family protein | probably involved in lipid binding (Kinch et al. 2005) |
| 568124-569689 | TTC0586 | phosphoglycerate dehydrogenase | involved in glycolytic pathway |
| 569809-570243 | TTC0587 | MarR family transcriptional regulator | transcriptional regulator |
| 570390-571565 | TTC0588 | mannosyl-3-phosphoglycerate synthase | glycosyltransferase involved in the two-step synthetic pathway of mannosylglycerate |
| 571562-572341 | TTC0589 | mannosyl-3-phosphoglycerate phosphatase | phosphorylation of mannosylglycerate |
| 571313-573809 | TTC0590 | PucR family transcriptional regulator | transcriptional regulator |
| 573834-574733 | TTC0591 | dihydrodipicolinate synthase | involved in the synthesis of dihydrodipicolinate |
| 574730-575470 | TTC0592 | 2-hydroxyhepta-2,4-diene-1,7-dioate isomerase/5-carboxymethyl-2-oxo-hex-3-ene-1,7-dioate decarboxylase | 4-hydroxyphenylacetate degradation bifunctional  isomerase/decarboxylase |
| 575467-577014 | TTC0593 | 5-carboxymethyl-2-hydroxymuconate semialdehyde dehydrogenase | involved in the 4-hydroxyphenylacetate meta-cleavage pathway |
| 577027-578472 | TTC0594 | 4-hydroxyphenylacetate 3-monooxygenase | involved in the initial step of the 4-hydroxyphenylacetate degradation pathway |
| 578469-578918 | TTC0595 | probable oxidoreductase | involved in oxidation-reduction reactions |
| 578906-579865 | TTC0596 | homoprotocatechuate 2,3-dioxygenase | a dioxygenase with catalase activity |
| 579963-581135 | TTC0597 | leucine-, isoleucine-, valine-, threonine-, and alanine-binding protein | amino acid-binding protein |
| 581139-582116 | TTC0598 | branched-chain amino acid transport system permease protein livH | a permease protein involved in branched-chain amino acid transportation |
| 582113-583870 | TTC0599 | branched-chain amino acid transport system permease protein livM/branched-chain amino acid transport ATP-binding protein livG | a permease protein involved in branched-chain amino acid transportation |
| 583867-584598 | TTC0600 | branched-chain amino acid transport ATP-binding protein livF | involved in branched-chain amino acid transportation |
| 584595-585005 | TTC0601 | probable phenylacetic acid degradation protein | probably involved in phenylacetic acid degradation |
| 584959-586296 | TTC0602 | phenylacetyl-CoA ligase | involved in the catabolism of phenylacetic acid |
| 586299- 586724 | TTC0603 | thioesterase family protein | catalytic termination of fatty acid chains |
| 588774-586768 | TTC0604 | phenylacetic acid degradation protein PaaZ | involved in phenylacetic acid degradation |

* The loci names, gene products and potential functions of the proteins are essentially based on the *T. thermophilus* HB27 genome annotations (Henne et al. 2004)

**Supplementary Fig. 1**


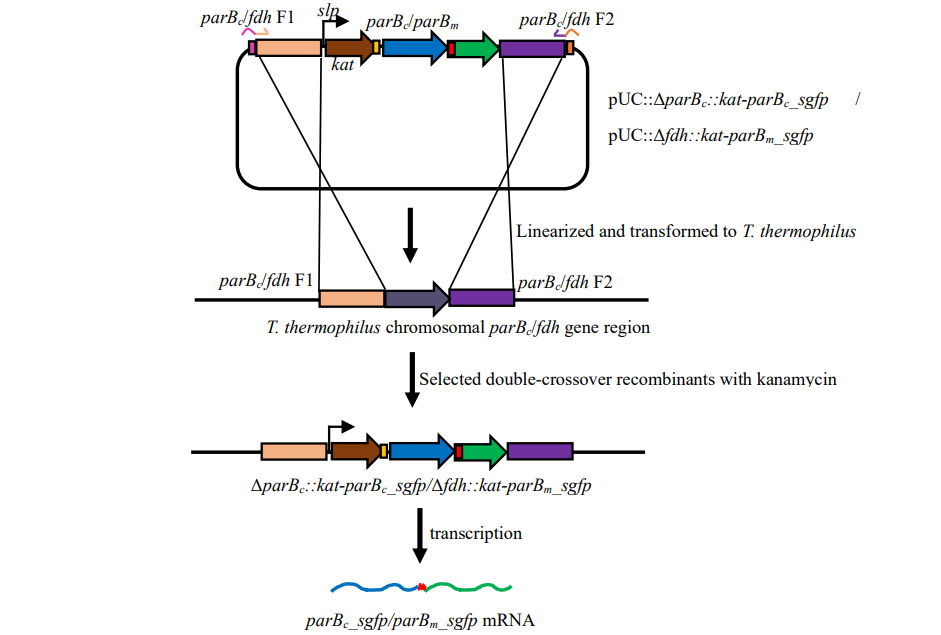

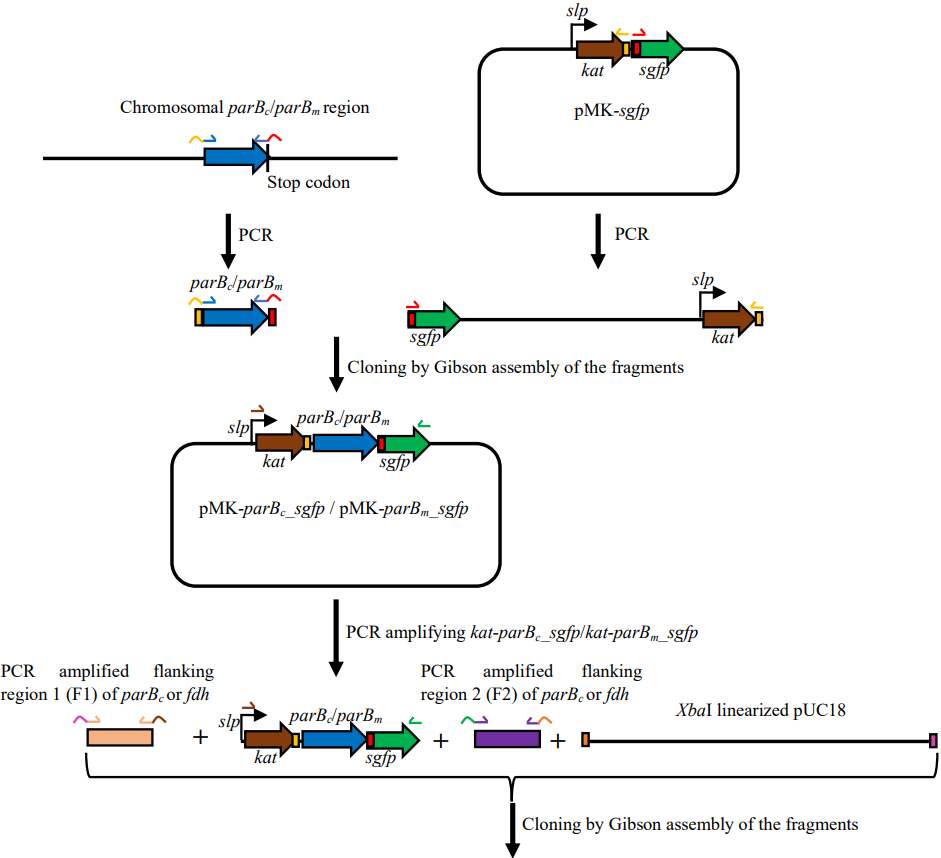


**Supplementary Fig. 1** Schematic illustrations of the cloning procedures of the constructs pUC::Δ*parB_c_::kat-parB_c__sgfp* and pUC::Δ*fdh::kat-parB_m__sgfp*, in which, *parB_c_* or *parB_m_* were translationally fused with *sgfp*. The short colored arrows (or with arcs) indicate primers; the small red, yellow, orange and pink boxes indicate primer binding sequences. Correspondingly, the red, yellow, orange and pink arcs in the primers indicate 20-bp sequences in the 5’ end, allowing for generation of PCR products with 20-bp overlapping sequences to the target fragments, thus the clonings could be accomplished by the Gibson assembly method.


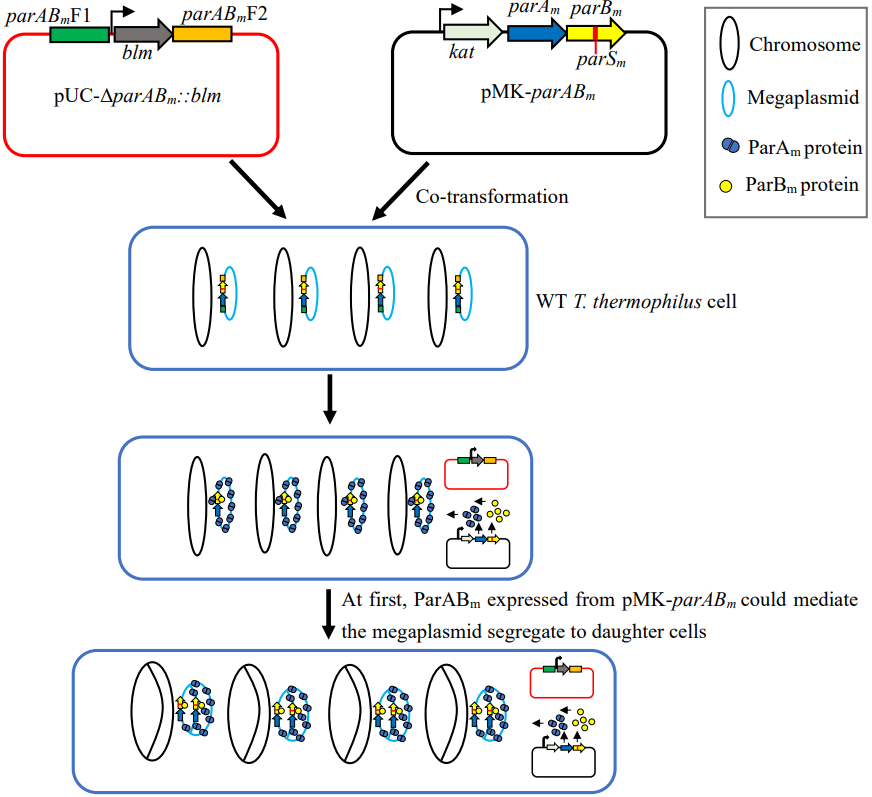

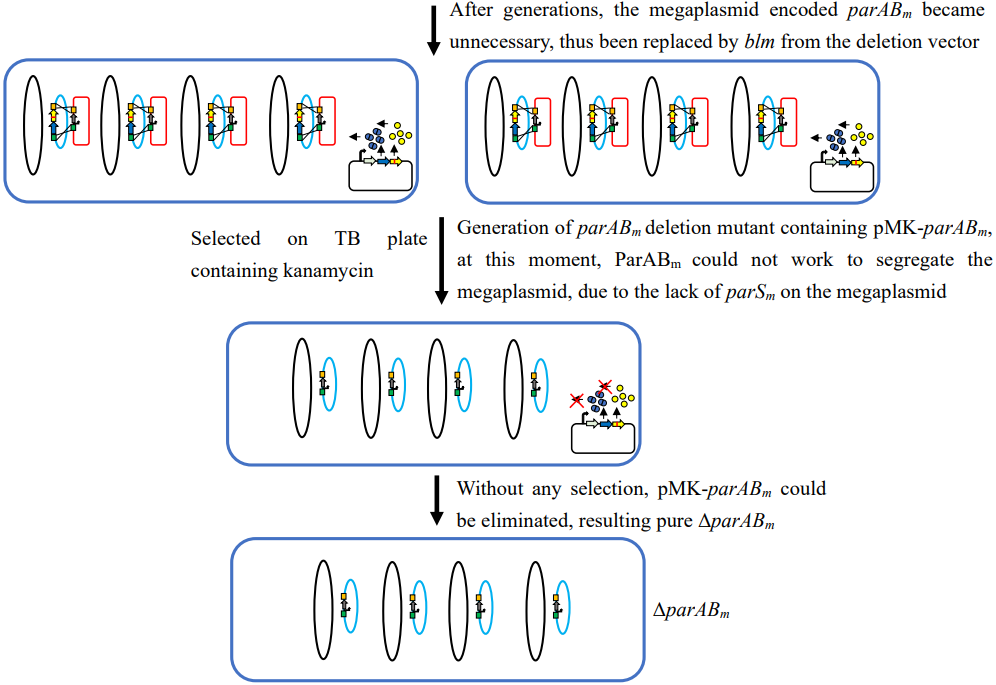


**Supplementary Fig. 2**

**Supplementary Fig. 2** Schematic illustrations of the generation principle and procedure of the Δ*parAB_m_* mutant. For simplicity, four copies of the chromosome and megaplasmid are shown, respectively. The space arrangement of the genome was hypothesized from previous studies (Li et al. 2015; Watanabe 2020). *parAB_m_*F1 (green box) and *parAB_m_*F2 (yellow box) indicate the two flanking regions (around 1kb) of the *parAB_m_* genes. The detailed description of the mutant generation principle and procedure are introduced in the “materials and methods” of the main text, and also in the embedded textboxes of the figure.

**Supplementary Fig. 3**


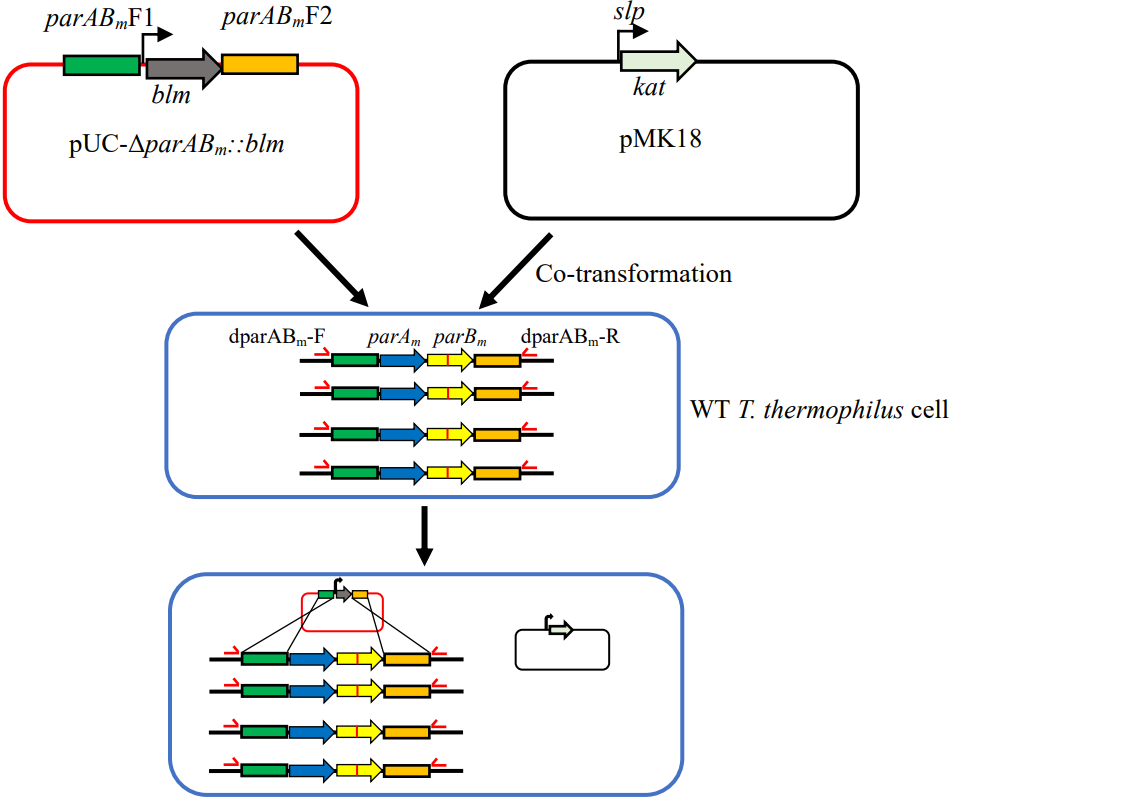

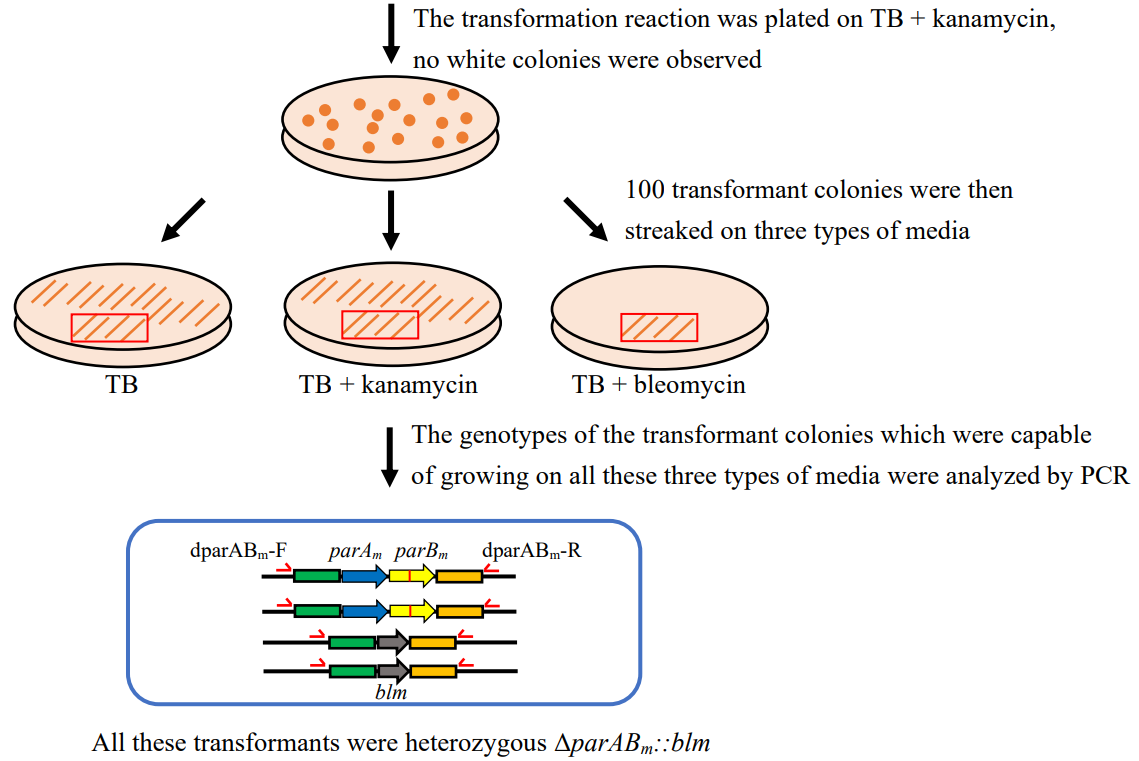


**Supplementary Fig. 3** Schematic illustrations of the co-transformation procedure and result of pUC-Δ*parAB_m_::blm* and pMK18 to the WT *T. thermophilus* cells. For simplicity, in the *T. thermophilus* cell, only four copies of the megaplasmid *parAB_m_* regions are shown. *parAB_m_*F1 (green box) and *parAB_m_*F2 (yellow box) indicate the two flanking regions (around 1 kb) of the *parAB_m_* genes (blue and yellow arrows, respectively). The two short red arrows indicate primer pairs dparAB_m_-F and dparAB_m_-R (Supplementary Table 1), which were used to confirm the heterozygous Δ*parAB_m_::blm* mutant by PCR.

**Supplementary Fig. 4**


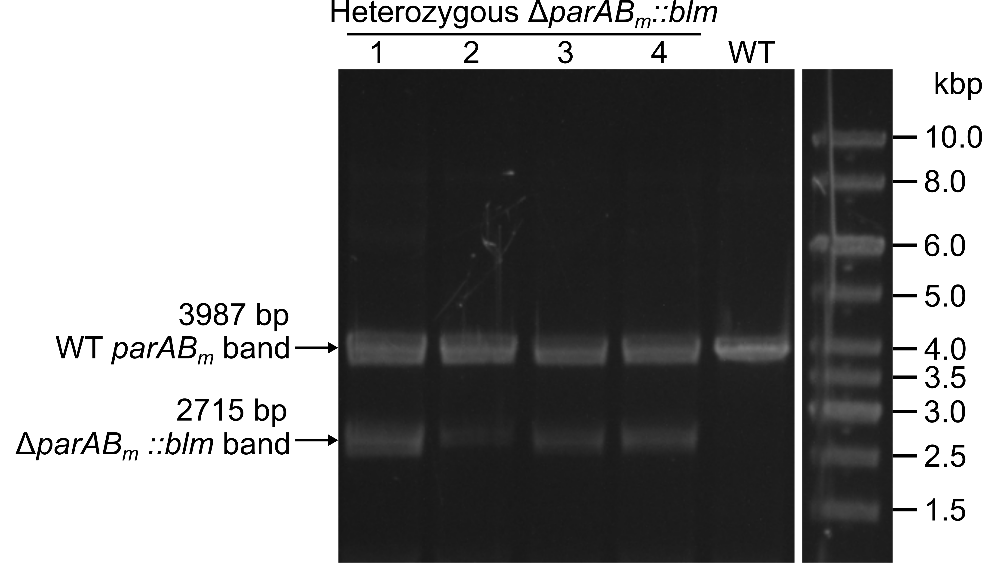


**Supplementary Fig. 4** The co-transformation result of pUC-Δ*parAB_m_::blm* and pMK18 to the WT *T. thermophilus* cells. The mutants were confirmed by PCR using two primers (dparAB_m_-F and dparAB_m_-R) flanking the two homology regions of *parAB_m_*. 1, 2, 3, 4 are four transformant colonies which were capable of growing on the three types of plates, i.e., TB, TB + kanamycin and TB +bleomycin (see Supplementary Fig. 3). The PCR result showed that the four transformants were all heterozygous containing both WT-*parAB_m_* and Δ*parAB_m_::blm* alleles. The 1 kb DNA marker (from Thermo Fisher Scientific) was used for the agarose gel electrophoresis.

**Supplementary Fig. 5**


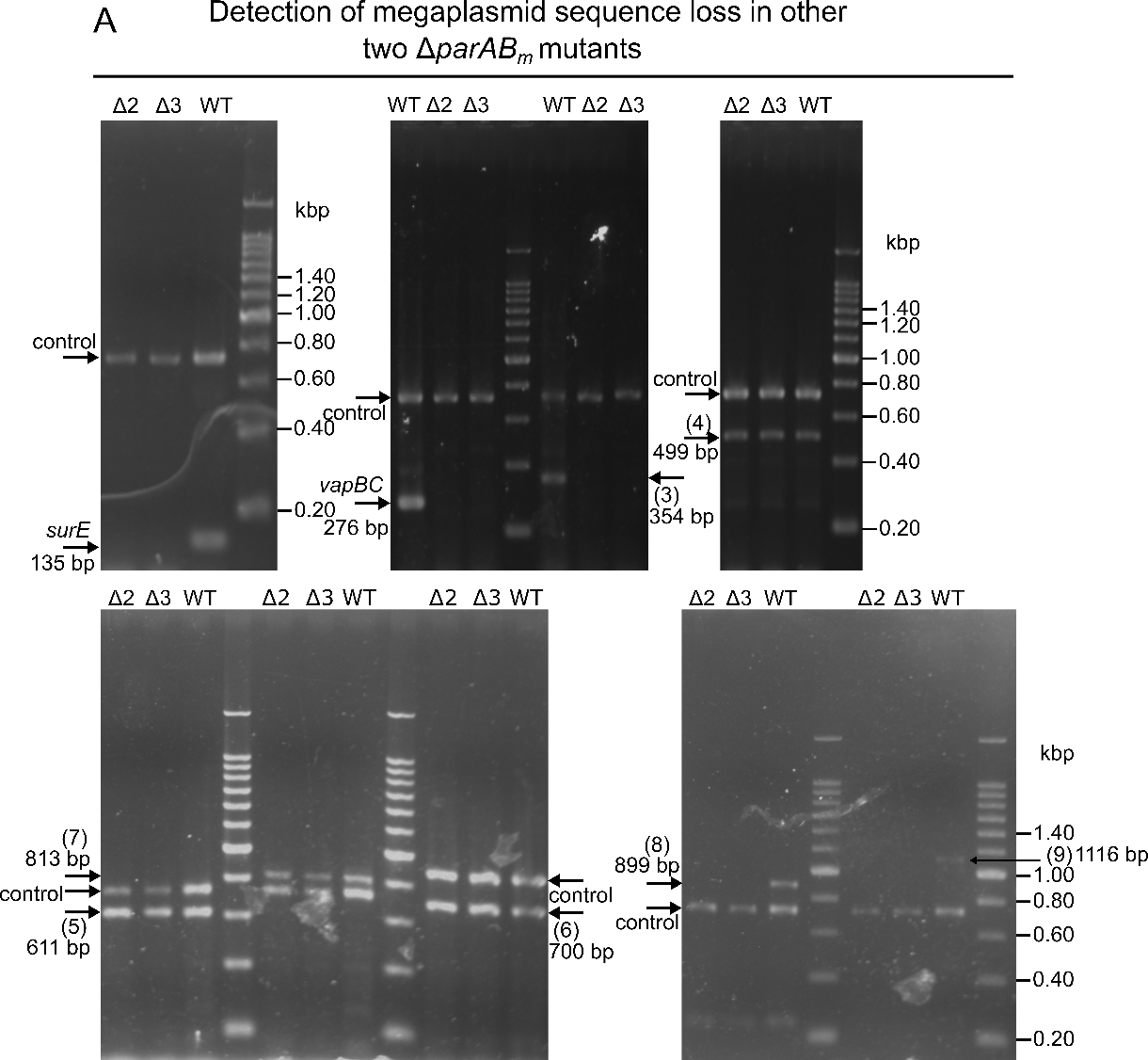


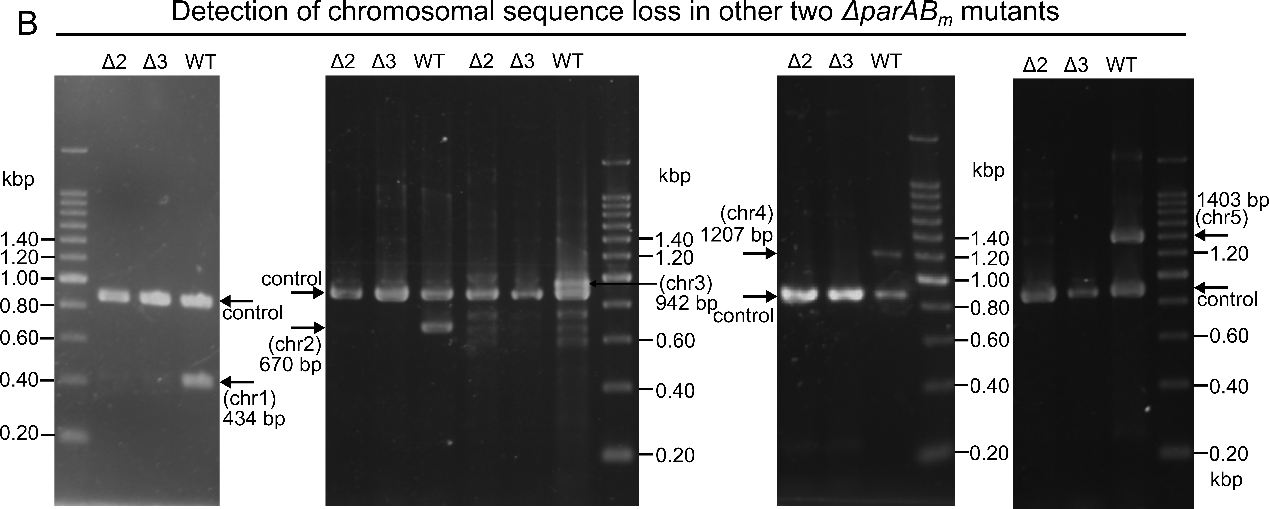


**Supplementary Fig. 5** PCR confirming the megaplasmid (A) and chromosomal (B) sequence loss in the other two Δ*parAB_m_* mutants. In each reaction, two sets of primers were used, which could bind the control gene region [TTC0825 (734 bp) or TTC1220 (890 bp) for detecting megaplasmid deletion, and TTC0439 (857 bp) for detecting chromosome deletion] and the target amplification region, respectively. Δ2 and Δ3 indicate the two Δ*parAB_m_* mutants. The genomic positions of the target amplicons are indicated in Fig. 3d.

**Supplementary Fig. 6**


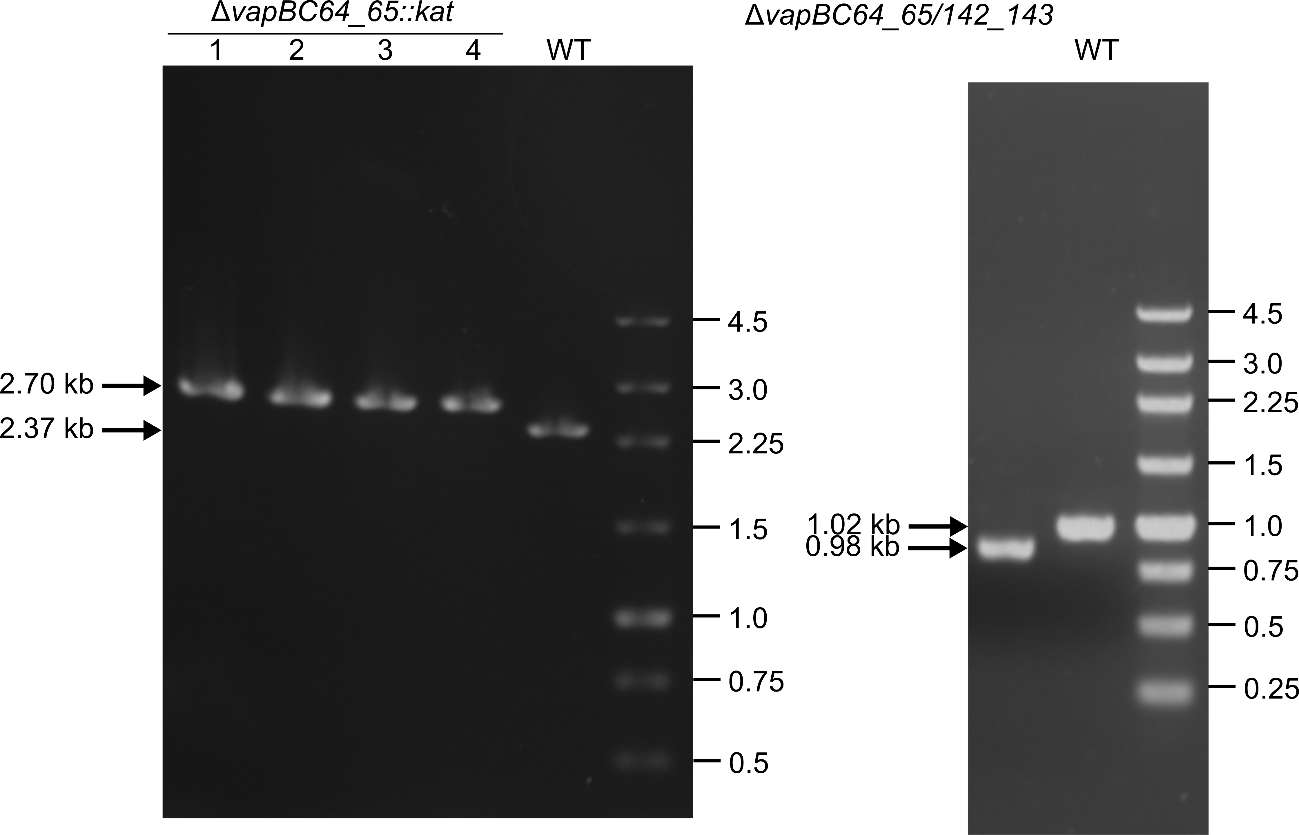


**Supplementary Fig. 6** Generation of the *T. thermophilus* Δ*vapBC64_65::kat* and Δ*vapBC64_65/142_143* mutants. The mutants were confirmed by PCR using primers binding the two flanking regions of the *vapBC64_65* genes (left) or *vapBC142_143* (right) genes, respectively. 1, 2, 3, 4 are four individual Δ*vapBC64_65::kat* colonies.

**Supplementary Fig. 7**


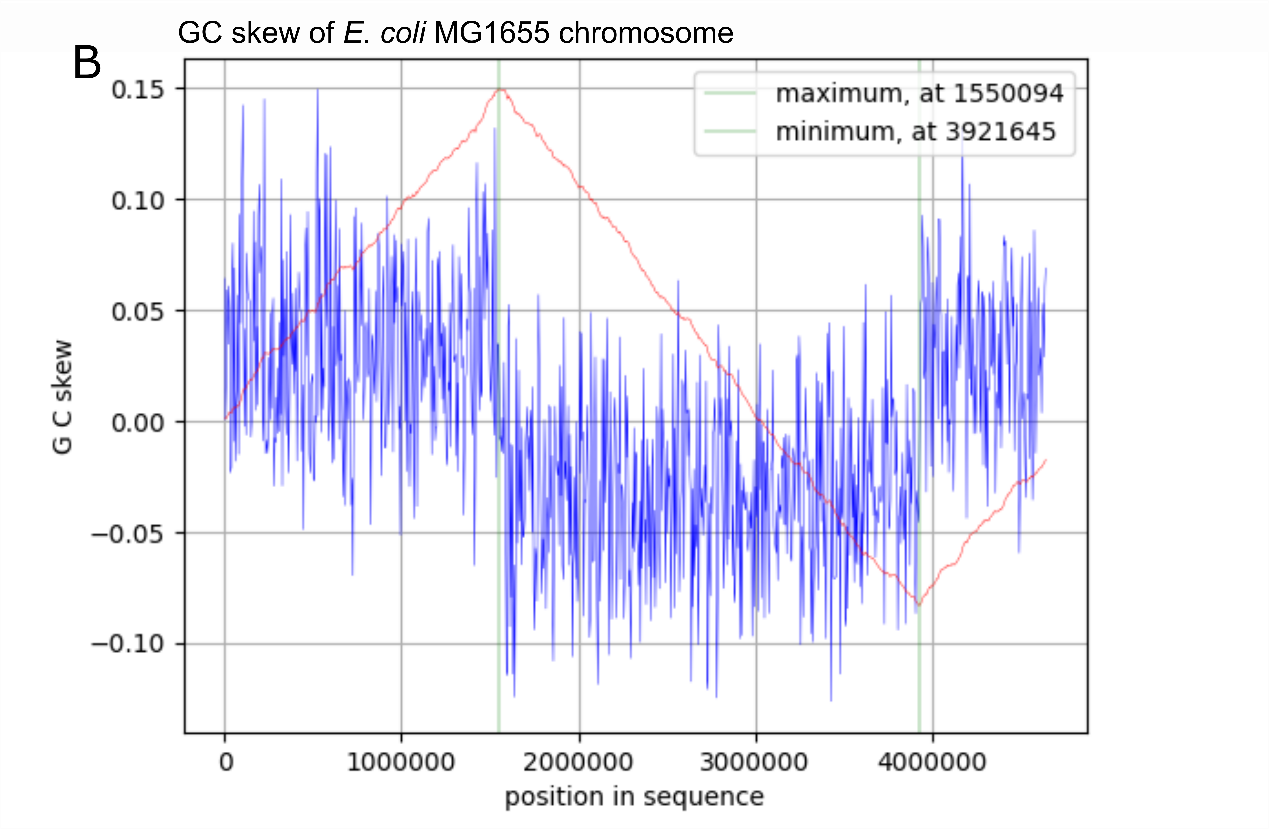

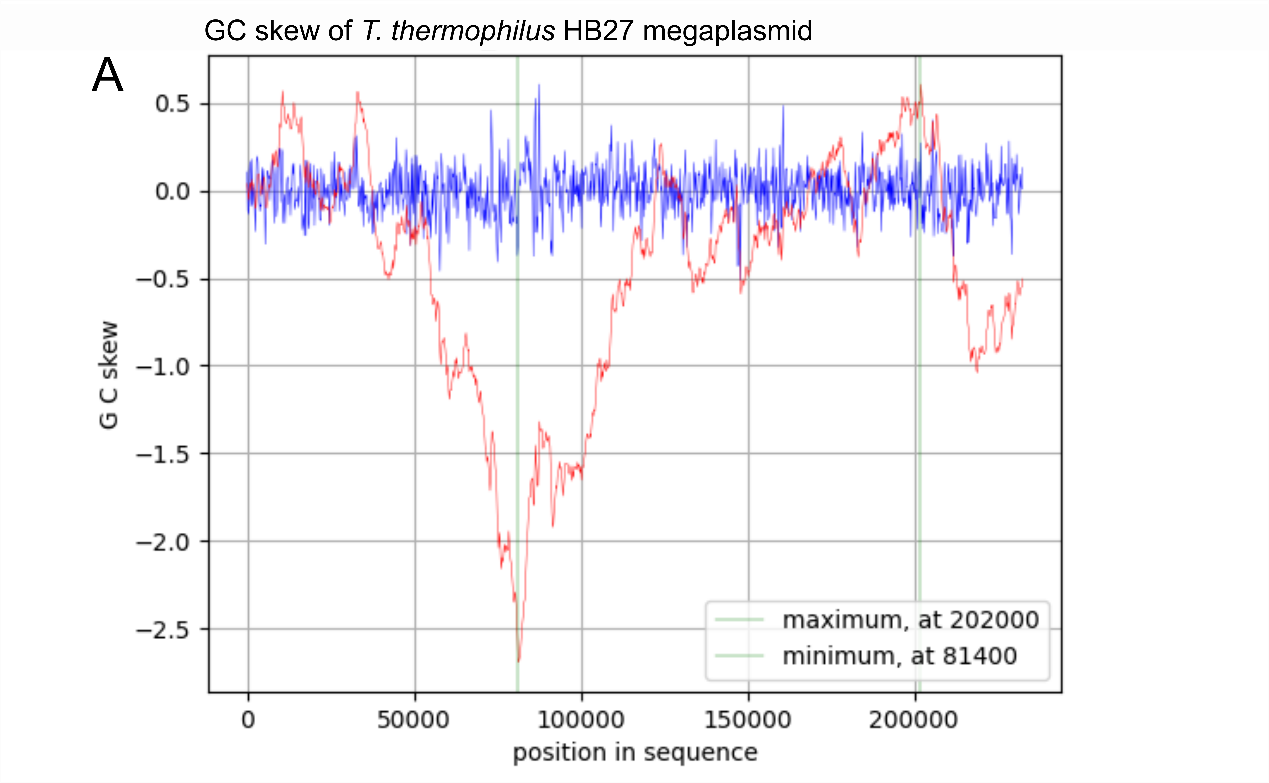


**Supplementary Fig. 7**  Comparison of the GC skew results between the *T. thermophilus* HB27 megaplasmid (A) and the *E. coli* MG1655 chromosome (B). The GC skew method is based on the principle that the nucleotide contents of the leading and lagging DNA strands are different (i.e., skew calculated as [G-C]/[G+C]) (Grigoriev 1998). The online version (<https://genskew.csb.univie.ac.at/webskew#usage_webskew>) of the Geneskew software was used. For the *T. thermophilus* HB27 megaplasmid sequence (NC_005838.1), the windowsize and stepsize were set as 200 and 210, respectively; for the *E. coli* MG1655 chromosome (NC_002695.2), the windowsize and stepsize were set as 4641 and 4641, respectively. The minimum indicates the predicted *oriC* region, the maximum indicates the predicted *ter*. In the *T. thermophilus* HB27 megaplasmid GC skew, many high-G/high-C shift points could be detected.

**Supplementary Fig. 8**


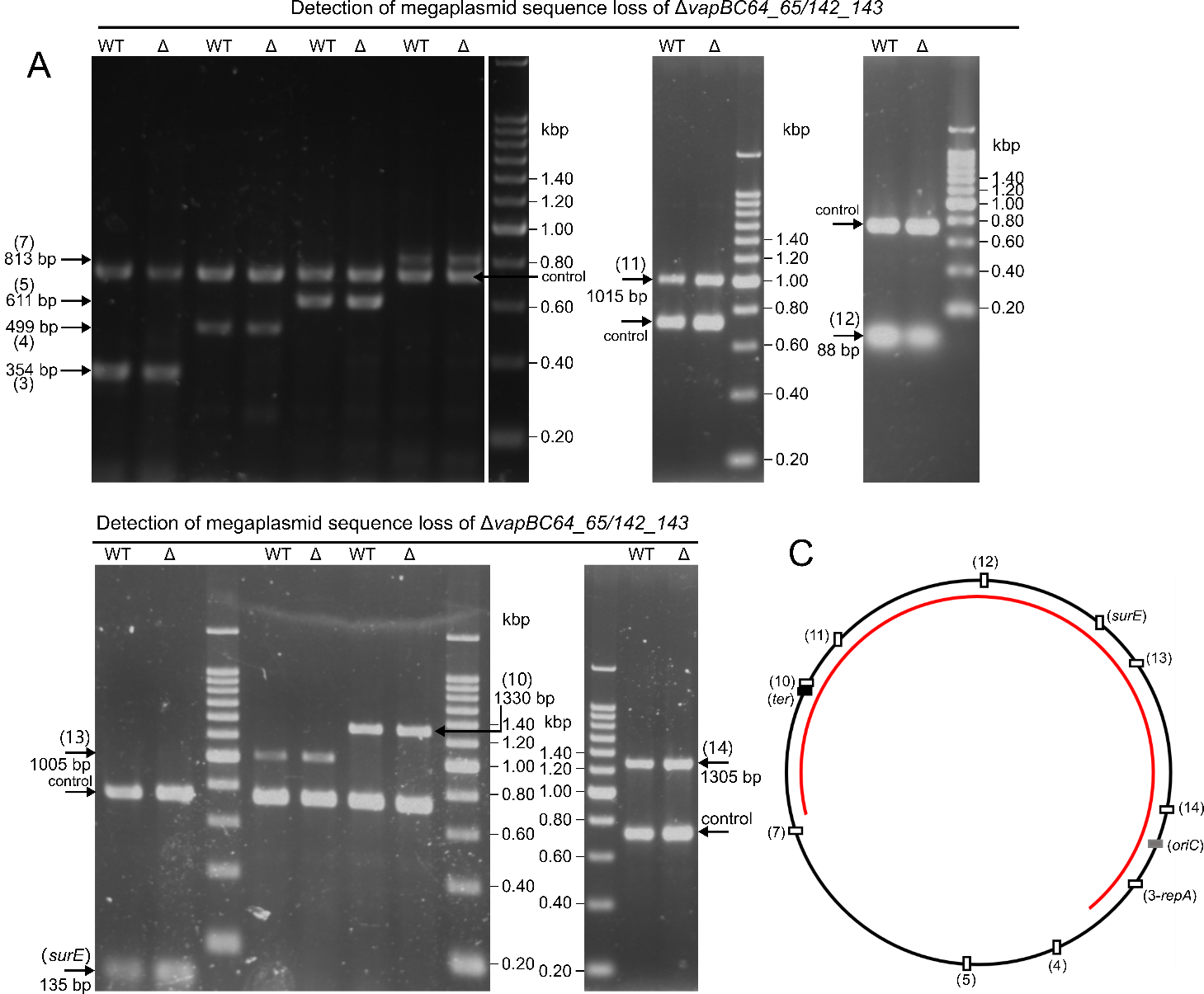


**
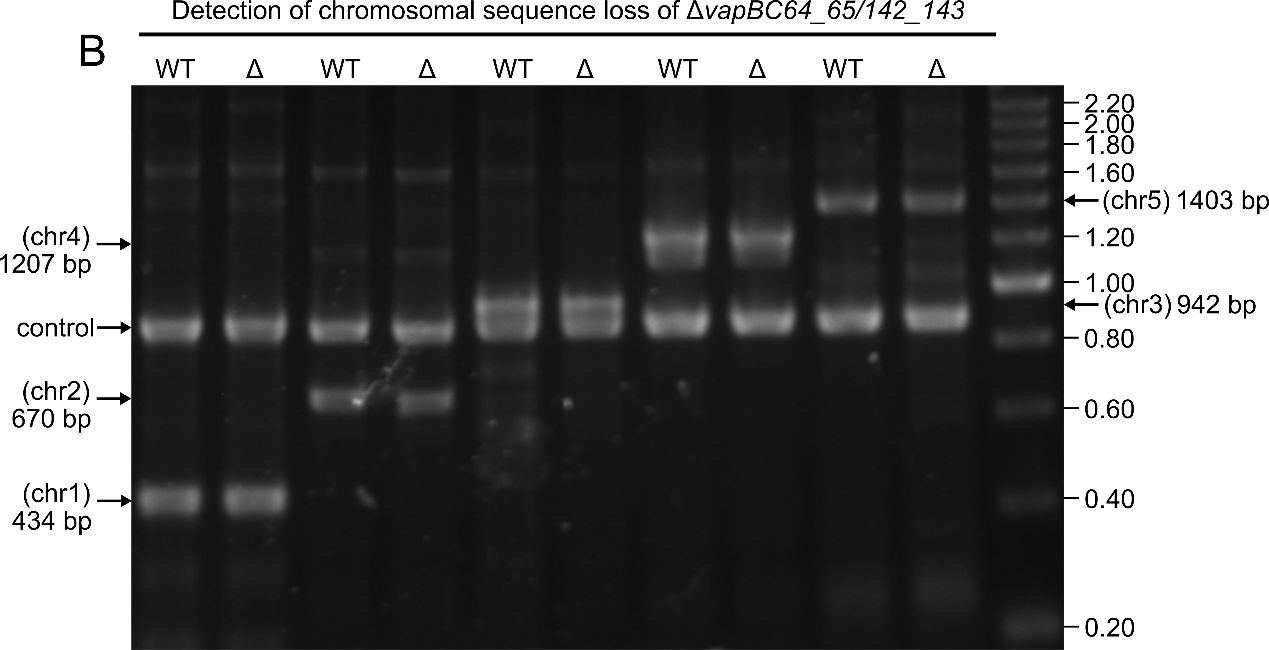
**

**Supplementary Fig. 8** PCR was used to analyze whether megaplasmid (A) and chromosomal (B) sequences were deleted in the Δ*vapBC64_65/142_143* mutant. In each reaction, two sets of primers were used, which could bind the control gene region [i.e., TTC0825 (734 bp) for detecting megaplasmid deletion, TTC0439 (857 bp) for detecting chromosome deletion] and the target amplification region, respectively. The genomic positions of the target megaplasmid amplicons are indicated in C (small white boxes, the positions are consistent with those indicated in Fig. 3d), and those of the chromosomal amplicons are indicated in Fig. 3d. The small gray and black boxes represent the origin and terminus regions of the megaplasmid. The red arc in C indicates the deletion region of the Δ*parAB_m_* mutant (also see Fig. 3d).

**Supplementary Fig. 9**


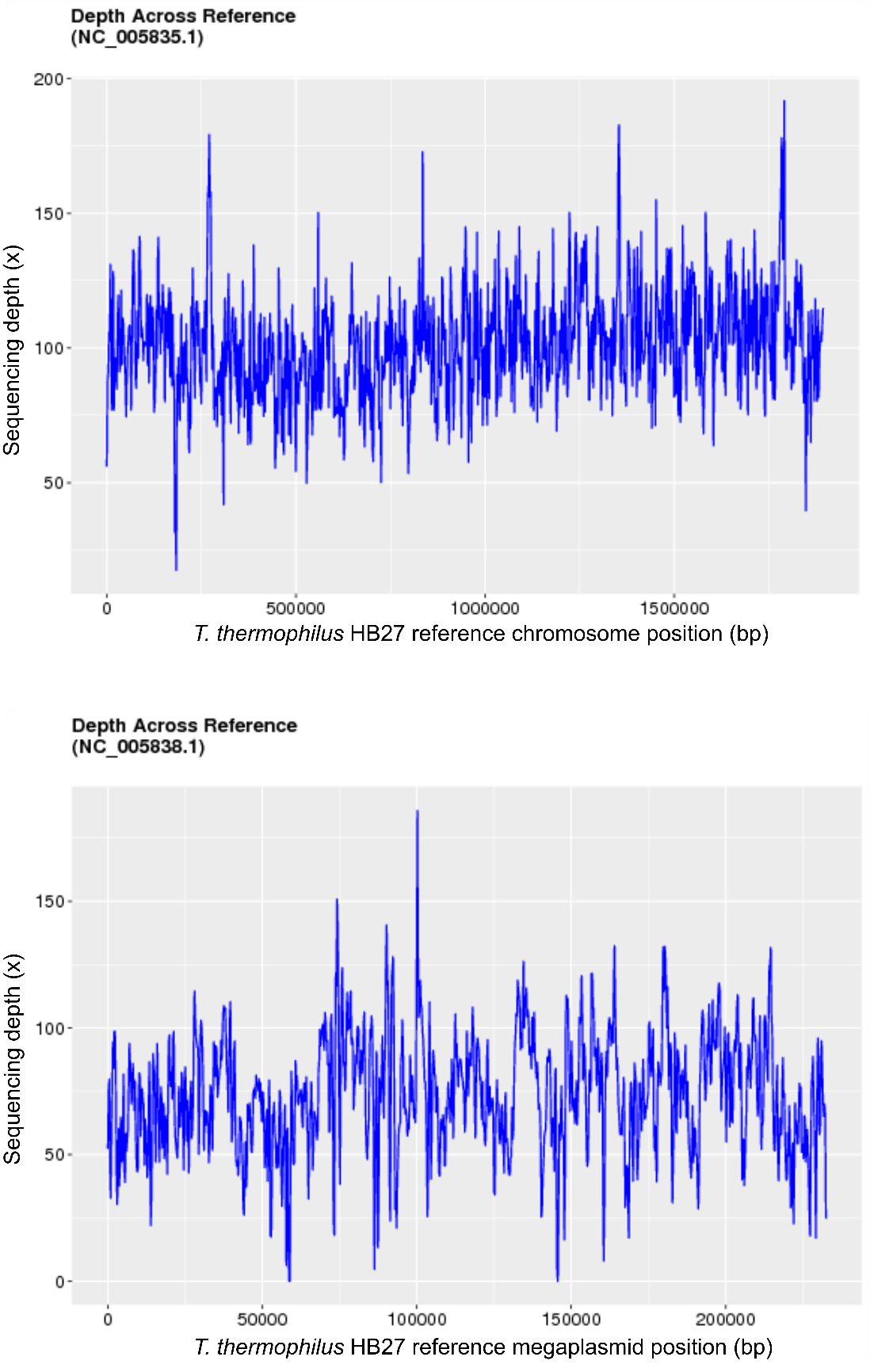


**Supplementary Fig. 9** Whole genome sequencing result of the Δ*vapBC64_65*/*142_143* mutant. Shown is the alignment of the Illumina reads to the reference genome (upper panel: chromosome, lower panel: megaplasmid) of *T. thermophilus* HB27 (chromosome 1,894,877 bp, megaplasmid 232,605bp, NCBI reference sequence: GCA_000008125.1). In total 1,418,324 clean reads (212,748,600 bp) were obtained for the Δ*vapBC64_65*/*142_143* mutant sequencing, the average sequencing depth was 96.91 (×) and the coverage rate was 99.94%.

**Supplementary references**

Grigoriev A. Analyzing genomes with cumulative skew diagrams. Nucleic Acids Res. 1998;26(10):2286-90. doi: 10.1093/nar/26.10.2286.

Henne A, Brüggemann H, Raasch C, Wiezer A, Hartsch T, Liesegang H, Johann A, Lienard T, Gohl O, Martinez-Arias R, Jacobi C, Starkuviene V, Schlenczeck S, Dencker S, Huber R, Klenk HP, Kramer W, Merkl R, Gottschalk G, Fritz HJ. The genome sequence of the extreme thermophile *Thermus thermophilus*. Nat Biotechnol. 2004;22(5):547-553. doi: 10.1038/nbt956.

Kinch LN, Cheek S, Grishin NV. EDD, a novel phosphotransferase domain common to mannose transporter EIIA, dihydroxyacetone kinase, and DegV. Protein Sci. 2005;14(2):360-367. doi: 10.1110/ps.041114805.

Li H, Angelov A, Pham VT, Leis B, Liebl W. Characterization of chromosomal and megaplasmid partitioning loci in *Thermus thermophilus* HB27. BMC Genomics. 2015;16(1):317. doi: 10.1186/s12864-015-1523-3.

Watanabe S. Cyanobacterial multi-copy chromosomes and their replication. Biosci Biotechnol Biochem. 2020;84(7):1309-1321. doi: 10.1080/09168451.2020.1736983.
